# A putative *R3 MYB* repressor is the candidate gene underlying *atroviolacium*, a locus for anthocyanin pigmentation in tomato fruit

**DOI:** 10.1093/jxb/erx382

**Published:** 2017-11-25

**Authors:** Xue Cao, Zhengkun Qiu, Xiaotian Wang, Tong Van Giang, Xiaolin Liu, Jing Wang, Xiaoxuan Wang, Jianchang Gao, Yanmei Guo, Yongchen Du, Guoping Wang, Zejun Huang

**Affiliations:** 1 The Key Laboratory of Biology and Genetic Improvement of Horticultural Crops of the Ministry of Agriculture, Institute of Vegetables and Flowers, Chinese Academy of Agricultural Sciences, China; 2 Department of Vegetable Science, College of Horticulture, South China Agricultural University, China

**Keywords:** Anthocyanin, *atroviolacium*, fine-mapping, MYB repressor, SlMYBATV, transcriptional analysis, tomato

## Abstract

Anthocyanins are potential health-promoting compounds in the human diet. The *atv* (*atroviolacium*) locus, derived from the wild tomato species *Solanum cheesmaniae*, has been shown to enhance anthocyanin pigmentation in tomato fruit when it co-exists with either the *Aft* (*Anthocyanin fruit*) or the *Abg* (*Aubergine*) locus. In the present study, the *atv* locus was fine-mapped to an approximately 5.0-kb interval on chromosome 7. A putative *R3 MYB* repressor was identified in this interval and is hereby designated as *SlMYBATV*. The allele of *SlMYBATV* underlying the *atv* locus harbored a 4-bp insertion in its coding region, which is predicted to result in a frame-shift and premature protein truncation. The other candidate R3 MYB and R2R3 MYB repressors of anthocyanin biosynthesis were also identified in tomato via a genome-wide search. Transcriptional analysis showed that most of the structural genes and several regulatory genes of anthocyanin biosynthesis were up-regulated in the tomato *SlMYBATV* mutant lines. These findings may facilitate the elucidation of the molecular mechanisms underlying anthocyanin pigmentation in tomato fruit and help in the marker-assisted selection of anthocyanin-enriched tomato cultivars.

## Teaser

Fine-mapping of the *atv* locus identifies a putative R3 MYB repressor *SLMYBATV* as its candidate gene. We present a model of the anthocyanin gene regulation network in the peel of tomato fruit.

## Introduction

Anthocyanins belong to the bioactive family of compounds known as flavonoids. These play important biological roles in plants such as attracting pollinators and seed distributors, and providing protection against various stresses caused by pathogens, ultraviolet (UV), high-intensity light, wounding, cold temperature, and drought (Gould, 2004; Albert *et al.*, 2009; Olsen *et al.*, 2009; Zhang *et al.*, 2013). Anthocyanins are also the main pigments responsible for red, purple, and blue coloration in various vegetables and fruit, thereby contributing to their marketable quality (Barrett *et al.*, 2010; Jaakola, 2013). Anthocyanins are also recognized as compounds with potential health benefits in humans (He and Giusti, 2010; Li *et al.*, 2017).

The regulation of anthocyanin biosynthesis has been widely investigated in various plants, and it is known that two groups of genes are involved, namely structural and regulatory genes (Dooner *et al.*, 1991; Springob *et al.*, 2003; Koes *et al.*, 2005). Structural genes encode enzymes that directly participate in anthocyanin biosynthesis, and these include phenylalanine-ammonia lyase (*PAL*), 4-coumaryl:CoA ligase (*4CL*), chalcone synthase (*CHS*), chalcone isomerase (*CHI*), flavanone 3-hydroxylase (*F3H*), flavonoid 3′5′-hydroxylase (*F3′5′H*), dihydroflavonol 4-reductase (*DFR*), and anthocyanidin synthase (*ANS*) (Holton and Cornish, 1995; Springob *et al.*, 2003). Transcriptional regulation of anthocyanin biosynthesis is controlled by several classes of transcription factors (TFs), including MYB TFs, basic helix-loop-helix (bHLH) TFs, and WD-repeat (WDR) proteins. These TFs form a protein complex (MBW complex) that positively regulates the expression of structural genes (Bulgakov *et al.*, 2017; Xu *et al.*, 2015). In addition to the proteins that activate anthocyanin biosynthesis, two distinct groups of MYB TFs decrease anthocyanin production, namely R3-MYB and R2R3-MYB repressors, which respectively contain one or two repeats of the MYB domain region (Aharoni *et al.*, 2001; Dubos *et al.*, 2008; Matsui *et al.*, 2008; Zhu *et al.*, 2009; Paolocci *et al.*, 2011; Albert *et al.*, 2014).

Tomato (*Solanum lycopersicum*) is one of the most important vegetable crops in the world, and it is also a good model organism in plant molecular biology research (Kimura and Sinha, 2008). Anthocyanins are generally accumulated in tomato vegetative tissues. More than 20 anthocyanin mutants have been identified (Al-Sane *et al.*, 2011), and a few underlying genes have been identified by fine-mapping and map-based cloning: *anthocyanin absent* (*aa*), *anthocyanin free* (*af*), *anthocyanin reduced* (*are*), a*nthocyanin without* (*aw*), and *Hoffman’s anthocyaninless* (*ah*), which respectively encode a glutathione S-transferase (SlGSTAA), a chalcone isomerase (CHI), a flavonoid 3-hydroxylase (F3H), a dihydroflavonol 4-reductase (DFR), and a bHLH TF (SlAN1) (Goldsbrough *et al.*, 1994; Kang *et al.*, 2014; Maloney *et al.*, 2014; Qiu *et al.*, 2016; Zhang *et al.*, 2016). A high anthocyanin-accumulation line in a tomato T-DNA activation-tagging library has enabled the identification of an R2R3-MYB TF, anthocyanin 1 (ANT1), which shares high homology with AN2 from petunia (*Petunia hybrida*) (Mathews *et al.*, 2003). The other three R2R3-MYB TFs, SlAN2, SlAN2-like, and SlANT1-like, also enhance anthocyanin accumulation (Schreiber *et al.*, 2012; Kiferle *et al.*, 2015; Meng *et al.*, 2015).

Unfortunately, no anthocyanins are present in cultivated tomato fruit (Gonzali *et al.*, 2009). Three loci, *Anthocyanin fruit* (*Aft*), *atroviolacium* (*atv*), and *Aubergine* (*Abg*), enhance anthocyanin accumulation in fruit when they are introgressed into cultivated tomato from wild species (Rick *et al.*, 1994; Kendrick *et al.*, 1997; Jones *et al.*, 2003). The *Aft* genotype, which originates from the wild species *S. chilense*, is regulated by a single dominant gene (Jones *et al.*, 2003; Canady *et al.*, 2006). Recently, linkage analysis has shown that the *Aft* locus co-segregates with two R2R3-MYB TFs that are located on chromosome 10, *SlANT1* and *SlAN2*, both of which are involved in anthocyanin regulation in tomato (Mathews *et al.*, 2003; Boches and Myers, 2007; Sapir *et al.*, 2008; Kiferle *et al.*, 2015). The *Abg* locus, which originates from *S. lycopersicoides*, has also been mapped to chromosome 10 (Rick *et al.*, 1994; Canady *et al.*, 2006). Because they are located on the same chromosome arm, it is not clear whether *Abg* is an allele of *Aft*. The *atv* locus, which originates from *S. cheesmaniae*, enhances anthocyanin pigmentation in the entire plant, particularly in the vegetative tissues when it is introgressed into cultivated tomato (Rick *et al.*, 1968; Mes *et al.*, 2008). The amount of anthocyanins in tomato fruit dramatically increases when the *atv* locus is combined with either the *Aft* or the *Abg* locus (Mes *et al.*, 2008). Unlike the *Aft* and *Abg* loci, the *atv* locus follows a recessive pattern of inheritance and has been mapped to chromosome 7 (Rick *et al.*, 1968).

In the present study, the site of the *atv* locus was narrowed down to an approximately 5.0-kb interval on chromosome 7. Only one gene, *Solyc07g052490*, which putatively encodes an R3 MYB repressor, was identified in this interval and it is hereby designated as *SlMYBATV* (*Solanum lycopersicum MYB* at the *atv* locus). A 4-bp insertion was found in the transcripts of *SlMYBATV* underlying the *atv* locus, which is predicted to result in a frame-shift and premature protein truncation. The tomato lines harboring the *SlMYBATV* gene mutation not only exhibit increased expression of the structural genes, but there is also an influence on the expression of the regulatory genes of anthocyanin biosynthesis. The findings of the present study may facilitate the discovery of the molecular mechanisms underlying anthocyanin pigmentation in tomato fruit and help in marker-assisted selection of tomato breeds that produce high amounts of anthocyanin.

## Materials and methods

### Plant material

Tomato seeds were obtained from Johnny’s Selected Seeds (http://www.johnnyseeds.com/; variety Indigo Rose) and the Tomato Genetics Resource Center (http://tgrc.ucdavis.edu/; variety Heinz1706). Indigo Rose bears fruit that are purple-skinned when ripe and high in anthocyanins, and Heinz1706 bears red fruit (wild-type). The populations for gene mapping and gene expression analysis were derived from the cross between Indigo Rose and Heinz1706. The F_2_ population of 190 individuals was grown in a plastic greenhouse in Shunyi District, Beijing in the summer of 2015. Both the F_3_ and F_5_ populations were grown in Haidian District, Beijing. A total of 164 recombinants selected from 1900 seedlings of an F_3_ population were grown in a glass greenhouse in the autumn of 2015, and 12 *Aft*/*Aft ATV*/*ATV* (abbreviated as *Aft*/*Aft*) plants and 12 *Aft*/*Aft atv*/*atv* plants selected from 94 seedlings of an F_5_ population were grown in a plastic greenhouse in the autumn of 2016.

### Development of PCR-based markers

An insertion/deletion (InDel) in the *SlAN2* gene was identified through sequence comparison of the *SlAN2* gene between LA1996 (a tomato line containing the *Aft* locus) and Heinz1706. (the GenBank Accession Number of the *SlAN2* gene of LA1996 is FJ705320). The InDels and SNPs on chromosome 7 and 10 were identified through sequence comparisons between Heinz1706 and LA1589 (a wild tomato line, *S. pimpinellifolium*), LA0716 (a wild tomato line, *S. pennellii*), and Indigo Rose. The whole genomic sequences of Heinz1706, LA1589, and LA0716 are available at the Sol Genomics Network (SGN, https://solgenomics.net/). PCR primers matching the flanking regions of these InDels and SNPs were designed using the Primer-BLAST tool of the National Center for Biotechnology Information (NCBI; http://www.ncbi.nlm.nih.gov/tools/primer-blast/index.cgi?LINK_LOC=BlastHome), and the primers were then used to screen co-dominant markers between Heinz1706 and Indigo Rose.

The PCR conditions were as follows: 94 °C for 4 min; followed by 35 cycles of 94 °C for 30 s, 52 °C for 30 s, 72 °C for 30–50 s; 72 °C for 5 min; and final temperature of 16 °C. The PCR products were separated on a 3% agarose gel, except for the product of marker ATV-In, which was separated on a 8% polyacrylamide gel. For the markers of cleaved amplified polymorphic sequences (CAPS) or derived cleaved amplified polymorphic sequences (dCAPS), the PCR products were digested with 2 U of a restriction enzyme for 2 h at 37 °C. The digested products were separated on a 3% agarose gel. General information regarding the DNA markers used in this study is given in Supplementary Table S1 at *JXB* online.

### Genetic analysis and preliminary mapping

The F_2_ population was genotyped by using the InDel marker HP1953 in the *SlAN2* gene. The fruit color of the plants in the F_2_ population was determined at the fully ripened stage. The segregation ratio of the *atv* locus was tested by chi-squared analysis using 121 plants harboring the allele of the *SlAN2* gene from Indigo Rose in the F_2_ population. Four InDel markers on chromosome 7 were then used to genotype the 121 plants of the F_2_ population.

### Phenotypic analysis of the *atv* locus when combined with the *Aft* locus

Three plants that were homozygous for the *Aft* locus and heterozygous for the *atv* locus were selected from the F_2_ population for the generation of the F_3_ populations. In each F_3_ population, six plants that were homozygous for the *atv* mutant region, six plants that were homozygous for the *ATV* wild-type region, and five plants that were heterozygous for the *atv* region were selected and assessed according to two traits (fruit color and anthocyanin content) when the fruits were at the mature green and the fully ripened stages. Extraction and quantification of the anthocyanin were performed using methods described previously by Povero *et al.* (2011). Three biological replicates were collected for each genotype per fruit development stage, and each replicate consisted of at least five fruit from different plants.

### Fine-mapping

Approximately 1900 seedlings in the F_3_ population that were homozygous for the *Aft* locus were genotyped using the chromosome 7 markers HP1877, HP1885, and HP1917. A total of 164 recombinants that were determined to be homozygous as Indigo Rose for one or two of these markers were selected and propagated in the green house. These recombinants were further genotyped by using additional markers (see Supplementary Table S1) and examined for fruit color at the ripening stage.

### Sequence polymorphism analysis

The *SlMYBATV* cDNAs of Indigo Rose and Heinz1706 were obtained by 3′-RACE and reverse-transcription PCR (RT-PCR). 3′-RACE was performed by using a SMARTer RACE cDNA Amplification Kit (Cat. No. 634914, CLONTECH Laboratories, Inc.), and RT-PCR was performed by using Phusion High-Fidelity DNA Polymerase (Cat. No. M0530L, New England Biolabs) and primers for cDNA cloning (Supplementary Table S2). The amplified fragments were cloned by using a pEASY-Blunt Zero Cloning Kit (Cat. No. CB501-02, TransGen Biothech, Beijing, China). cDNA clones were sequenced at the Beijing Genomics Institute, Beijing, China.

The genomic fragments of the *SlMYBATV* gene of Indigo Rose were obtained by overlapping PCR, which was performed by using 2×Taq PCR mix (Beijing Emarbio Science & Technology Company, Beijing, China) and primers for sequencing (Supplementary Table S2). The amplified fragments were sequenced at the Beijing Genomics Institute, Beijing, China.

Sequence polymorphisms were identified by using BLASTn in the SGN and Clustal X ver. 2.1 (Larkin *et al.*, 2007).

### Phylogenetic tree construction

The protein sequences of the known R2R3 MYB repressors AtMYB4 (*Arabidopsis thaliana* MYB4, accession number Q9SZP1) (Kranz *et al.*, 1998), FaMYB1 (*Fragaria* × *ananassa* MYB1, AAK84064) (Aharoni *et al.*, 2001), PhMYB27 (*Petunia* × *hybrida* MYB27, AHX24372) (Albert *et al.*, 2011) and the known R3 MYB repressors AtCPC (*A. thaliana* CAPRICE, BAA21917) (Wada *et al.*, 1997), and PhMYBx (AHX24371) (Kroon, 2004) were downloaded from GenBank. Using the full-length protein sequences of these MYB repressors as queries, tomato homologs were identified by BLASTP in the database of Tomato Genome Protein Sequences (ITAG release 2.40) in the SGN. The candidate tomato R2R3 MYB repressors were identified by meeting two conditions: BLASTP with *E*-value ≤1 × 10^–60^ and containing a C2/EAR motif. The candidate tomato R3 MYB repressors were identified by meeting two conditions: BLASTP with *E*-value ≤1 × 10^–10^ and containing a single MYB domain.

Multiple alignment of the R2R3 MYB and R3 MYB domain sequences of SlMYBATV and the other MYB repressors (listed in Supplementary Table S3) was performed by Clustal X ver. 2.1 (Larkin *et al.*, 2007) using the default settings. The alignment results were imported into MEGA6 (Tamura *et al.*, 2013). An unrooted phylogenetic tree was constructed by using the UPGMA method, Jones–Taylor–Thornton (JTT) model, and 1000 replicates.

### Total RNA isolation, cDNA synthesis, and real-time PCR analysis

Samples of the peel (outer skin) were collected from mature green and fully ripened fruit on the same day, and rapidly frozen in liquid nitrogen. Each tissue sample comprised three biological replicates, each replicate contained samples from at least five fruit, and each fruit of a biological replicate was picked from a different plant. Total RNA was isolated by using a Huayueyang Quick RNA isolation Kit (Cat. No. ZH120, Huayueyang Biotechnology, Beijing, China), and cDNA was synthesized from 1 μg total RNA by using GoScript^TM^ Reverse Transcriptase (Cat. No. A5003, Promega, USA).

Real-time quantitative PCR was conducted by using the GoScript^TM^ qPCR Master Mix (Cat. No. A6002, Promega, USA) and a LightCycler 480 detection system (Roche Diagnostics GmbH, Mannheim, Germany). The volume of the real-time PCR reaction was 15 μl, made up of 3 μl 0.1× cDNA dilution liquid as template, 0.35 μl of the forward primer (10 μmol l^–1^), 0.35 μl of the reverse primer (10 μmol l^–1^) (Supplementary Table S4), 7.5 μl of the GoScript^TM^ qPCR Master Mix, and 3.8 μl of ddH_2_O. The real-time PCR conditions were as follows: 95 °C for 10 min; followed by 45 cycles of 95 °C for 10 s, 57 °C for 20 s, and 72 °C for 20 s; 95 °C for 5 s; 65 °C for 1 min; and then 40 °C for 10 s. A tomato *ACTIN* (*Solyc03g078400*) gene was used as the reference, and all analyses were performed by using three technical replicates. The 2^−∆*C*T^ method was used to calculate relative gene expression, which was determined from real-time PCR experiments (Livak and Schmittgen, 2001). The data were analysed by one-way ANOVA, and differences between tomato lines were tested by using the Tukey honest significant difference multiple comparisons test (*P<*0.01).

### Accession numbers

Sequence data from this article can be found in the GenBank libraries under the following accession numbers: *SlMYBATV* (MF197509), *SlAN2* (MF197510 and MF197511), *SlAN2-like* (MF197512), *SlMYB76* (MF197513), *SlMYBATV-like* (MF197514), *SlMYBATV-X1* (MF197515 and MF197518), *SlMYBATV-X2* (MF197516 and MF197519), *SlMYBATV-X3* (MF197517 and MF197520), and *SlTRY* (MF197521).

## Results

### Genetic analysis and preliminary mapping of the *atv* locus

**Fig. 1.**
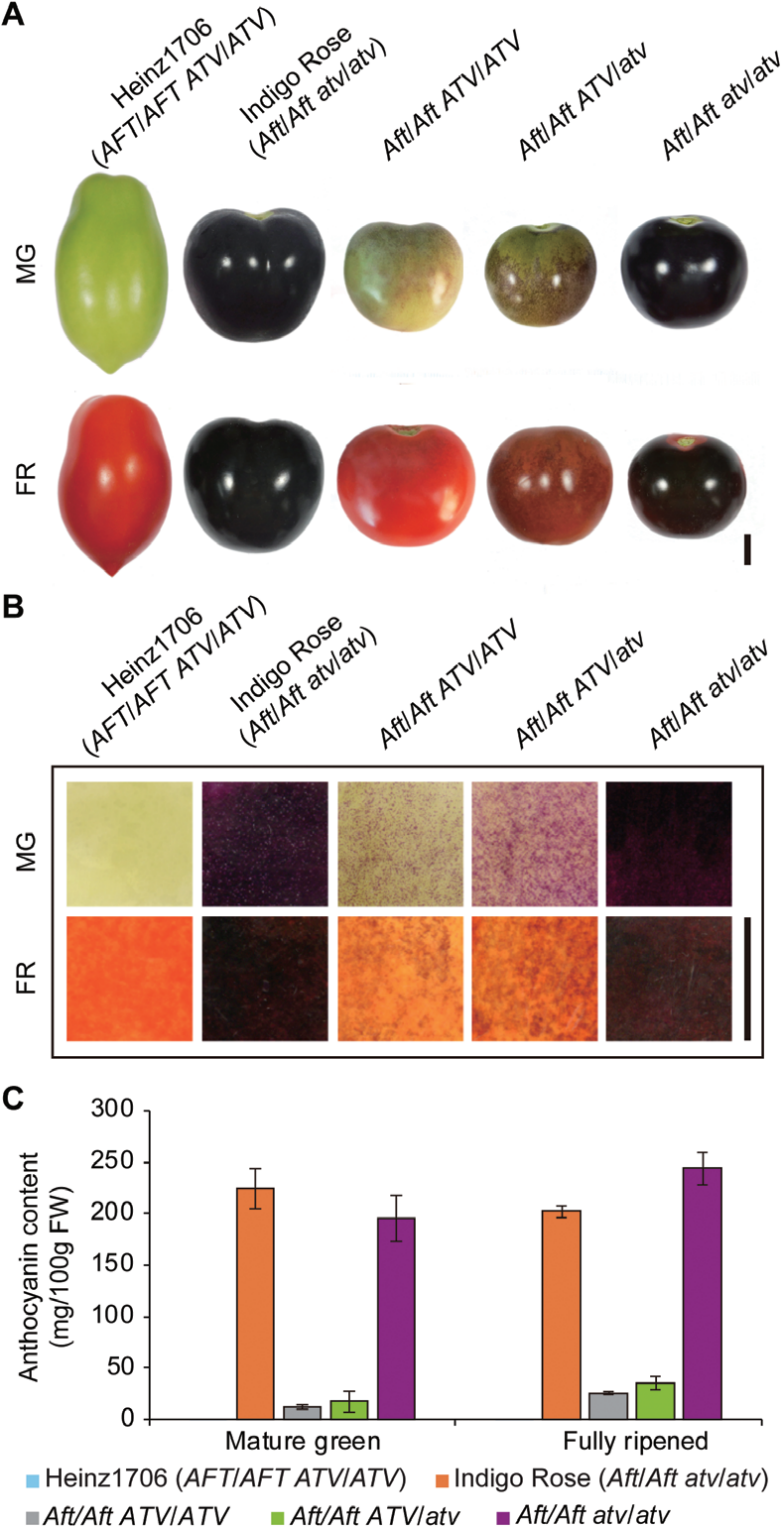
Anthocyanin accumulation in the fruit of different tomato genotypes. (A) Photographs of fruit at the mature green (MG) and fully ripened (FR) stages. (B) Scanned images of tomato peel taken from the fruit at the mature green and fully ripened stages. (C) Anthocyanin content in peel collected from fruit at the mature green and fully ripened stages. Heinz1706 is the wild-type, Indigo Rose is a variety containing both the *Aft* and *atv* loci. *AftAft ATVATV*, *AftAft ATVatv*, and *AftAft atvatv* are three genotypes of a F_3_ population that was derived from a cross between Heinz1706 and Indigo Rose. Scale bars in (A, B) indicate 1 cm.

The *atv* locus alone slightly increases anthocyanin pigmentation in fruit, so it is difficult to distinguish between the wild-type and *atv* mutant based on the fruit color. However, when the *Aft* locus is present, the *atv* locus can dramatically increase anthocyanin pigmentation (Mes *et al.*, 2008; Povero *et al.*, 2011). For preliminary mapping of the *atv* locus, the tomato variety Indigo Rose was selected because it contains both the *Aft* and *atv* loci (http://extension.oregonstate.edu/gardening/purple-tomato-debuts-indigo-rose) and bears fully purple-skinned ripe fruit (Fig. 1). To exploit the effect of the *Aft* locus on anthocyanin pigmentation, the individuals most likely containing it in the F_2_ population were identified by genotyping using an InDel marker HP1953 within the *SlAN2* gene, a candidate gene of *AFT* (Boches and Myers, 2007). A total of 121 plants were detected to harbor an allele of the *SlAN2* gene from Indigo Rose and all these plants bore purple fruit. This result suggested that the marker HP1953 was tightly linked with the *Aft* locus. However, the levels of purple color varied among the 121 plants: 97 bore fruit with only purple spots and 24 bore fully purple fruit, similar to Indigo Rose. This was in agreement with the expected segregation ratio of 3:1 using the chi-square test (χ^2^=0.93, less than χ^2^_0.05_=3.84), which indicated that the *atv* locus followed a recessive pattern of inheritance and controlled the level of purple coloration in the fruit of these 121 plants.

The 121 plants were then genotyped using the chromosome-7 InDel markers (Supplementary Table S1) because the *atv* locus is located on this chromosome (Rick *et al.*, 1968). Based on the genotypes and fruit color phenotypes, the *atv* locus mapped to the interval between marker HP1877 and HP1885 on the long arm of chromosome 7 (Supplementary Table S5).

### Phenotypic analysis of the *atv* locus when combined with the *Aft* locus

Three tomato plants were selected from the F_2_ population to analyse the phenotype of the *atv* locus when combined with the *Aft* locus through progeny testing. The three plants were homozygous for the *Aft* locus and heterozygous for the *atv* locus, which was confirmed by using the InDel markers HP1949 and HP3195 around the *MYB* gene cluster, which encompassed the *SlAN2* and *SlANT1* genes, the InDel marker HP1953 within the *SlAN2* gene, and the chromosome-7 InDel markers HP1877 and HP1885. The genotypes of the *atv* locus of these plants were identified by progeny testing using the InDel markers HP1877 and HP1885. The fruit of the plants showed purple coloration at and after the mature green stage. The *Aft*/*Aft ATV*/*ATV* (abbreviated as *Aft*/*Aft*) and *Aft*/*Aft ATV*/*atv* plants bore fruit with purple spots, whereas the *Aft*/*Aft atv*/*atv* plants bore fully purple fruit similar to that of Indigo Rose (Fig. 1A, B). Quantitative analysis of anthocyanins in the fruit peel showed that *Aft*/*Aft atv*/*atv* accumulated a higher amount of anthocyanins than the other two genotypes (Fig. 1C).

### Fine-mapping and candidate gene identification of the *atv* locus

**Fig. 2.**
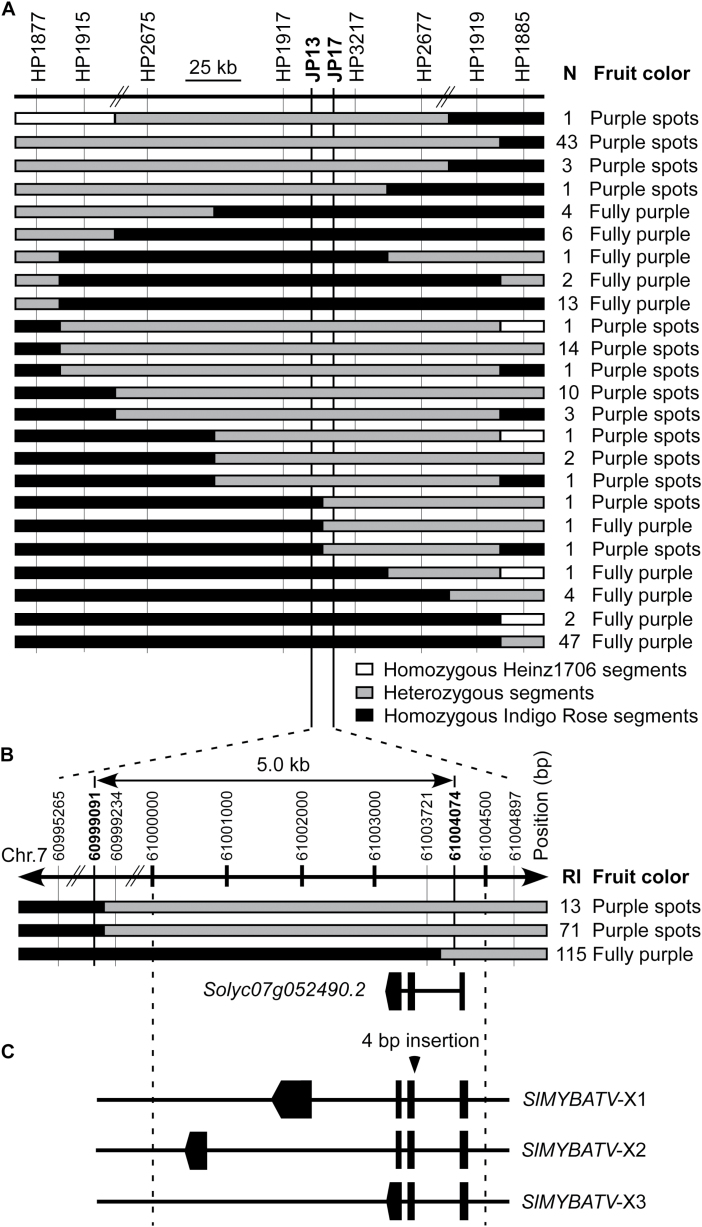
Fine-mapping of the *atv* locus. (A) The genotype and fruit color of the recombinants in the F_3_ populations. N indicates the number of recombinants. (B) Genotypes of three recombinants and the annotated gene according to ITAG release 2.40 at the *atv* locus. RI indicates the recombinant identification number. (C) The exon–intron structures of the *SlMYBATV* gene based on its three transcripts. Only the 4-bp insertion at the beginning of the second exon of the *SlMYBATV* mutant gene is shown. The other sequence polymorphisms are shown in Supplementary Figs. S1 and S2.

A total of 164 recombinants that contained homozygous Indigo Rose segments within the interval between markers HP1877 and HP1885 on chromosome 7 were selected from 1900 F_3_ individuals that were homozygous for the *Aft* locus. Their fruit color was assessed at the fully ripened stage. By analysing the genotypes and phenotypes of these recombinants, the *atv* locus was fine-mapped to an interval between markers JP13 and JP17 (Fig. 2A). The three recombinants whose cross-over sites were between these markers (Fig. 2A, B) were sequenced in this region to determine the precise position of the break-points. The break-points of the two recombinants that bore fruit with purple spots were between SL2.50ch07: 60999091 and SL2.50ch07: 60999234. Position SL2.50ch07: 60999091 and its upstream region were homozygous as the Indigo Rose parent line, whilst position SL2.50ch07: 60999234 and its downstream region were heterozygous. The break-point of the recombinant that bore fruit with fully purple color was between SL2.50ch07: 61003721 and SL2.50ch07: 61004074. Position SL2.50ch07: 61003721 and its upstream region were homozygous as the Indigo Rose parent line. Therefore, the position of the *atv* locus was narrowed down to an approximately 5.0-kb interval (SL2.50ch07: 60999091–61004074) on chromosome 7 (Fig. 2B).

Only one gene, *Solyc07g052490.2*, which putatively encodes an MYB transcription factor, was identified in this interval by using ITAG (International Tomato Annotation Group) ver. 2.40, which is the official annotation for the tomato genome (Fig. 2B). Based on this, *Solyc07g052490.2* was identified as the candidate gene of *ATV* and is hereby designated as *SlMYBATV* (*Solanum lycopersicum MYB* at the *atv* locus).

**Fig. 3.**
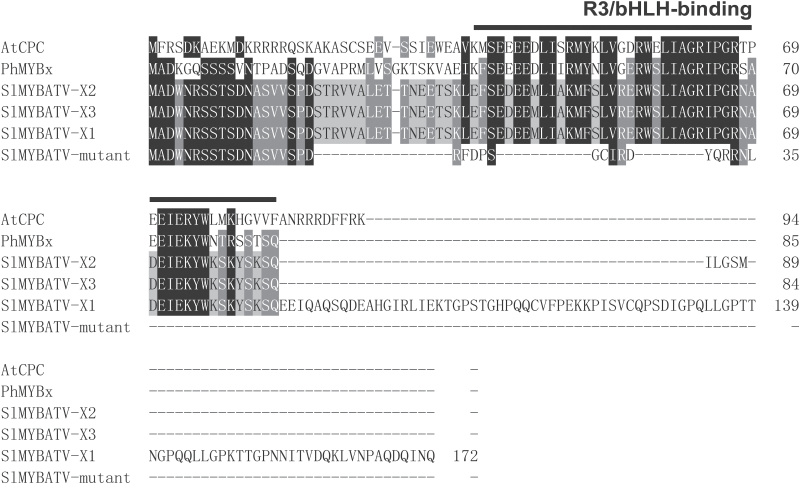
Multiple alignment of predicted protein sequences of SlMYBATV with AtCPC and PhMYBx. Entire protein sequences were aligned by using Clustal X ver. 2 (Larkin *et al.*, 2007) with default settings, and the conserved amino acids were shaded by using GeneDoc (2.6) (Nicholas *et al.*, 1997). The R3/bHLH-binding domain is marked above the alignment. ‘SlMYBATV-mutant’ indicates the deduced protein sequence from the transcripts of the purple-fruit tomato variety Indigo Rose.

The partial mRNAs of *SlMYBATV* from Heinz1706 were lengthened through 3′-RACE and RT-PCR. Three types of transcripts were identified and were designated as *SlMYBATV-X1*, *SlMYBATV-X2*, and *SlMYBATV-X3*, which were derived from four, four, and three exons, respectively. The first and the second exons were present in all these transcripts (Fig. 2C). The protein sequences, which were deduced from *SlMYBATV-X1*, *SlMYBATV-X2*, and *SlMYBATV-X3* from Heinz1706, comprised 172 amino acids (aa), 89 aa, and 84 aa, respectively. These deduced protein sequences of SlMYBATV were similar to the R3 MYB repressors AtCPC from *Arabidopsis* and PhMYBx from petunia (Kroon, 2004; Zhu *et al.*, 2009) and contained a R3/bHLH-binding domain (Fig. 3).

The three types of transcripts of *SlMYBATV* were also identified in Indigo Rose, but these all contained a 4-bp insertion that was located at the beginning of the second exon (Fig. 2C, Supplementary Fig. S1). This insertion was predicted to result in a frame-shift that would alter the protein sequences from amino-acid position 20 onwards by inducing premature termination of translation, including the R3/bHLH-binding domain (Fig. 3).

In addition to the 4-bp insertion, the *SlMYBATV* gene in Indigo Rose also harbored 47 SNPs, six small InDels (size ≤5 bp), one medium-sized InDel (size 22 bp), and one large InDel (size 367 bp) when compared to Heinz1706 (Supplementary Fig. S2). Among these, four SNPs and two small InDels (size 1 bp) were situated within the promoter and 5′- UTR region (Supplementary Fig. S2); however, these might not affect anthocyanin biosynthesis because they were not in the *atv* locus (Fig. 2B).

### Phylogenetic analysis of SlMYBATV and the MYB repressors for the phenylpropanoid and flavonoid pathways

The SlMYBATV protein of Heinz1706 contained one MYB/bHLH-binding domain and was similar to the R3 MYB repressors AtCPC and PhMYBx (Fig. 3 and Supplementary Fig. S3) (Kroon, 2004; Zhu *et al.*, 2009). Based on these findings, SlMYBATV is probably a MYB repressor because its loss of function is related to anthocyanin pigmentation in the fruit peel. MYB repressors play important roles in the regulation of phenylpropanoid and flavonoid biosynthesis (Xu *et al.*, 2015). Two other putative R3 MYB repressors, SlMYBATV-like (Solyc12g005800) and SlTRY (Solyc01g095640), have been identified in tomato, and four putative R2R3 MYB repressors for the phenylpropanoid and flavonoid pathway have also been identified: SlMYB3 (Solyc06g065100), SlMYB7 (Solyc01g111500), SlMYB32 (Solyc10g055410), and SlMYB76 (Solyc05g008250) (Zhao *et al.*, 2014). These harbor R2, R3/bHLH-binding, C1, and C2/EAR domains/motifs (Supplementary Fig. S3).

**Fig. 4.**
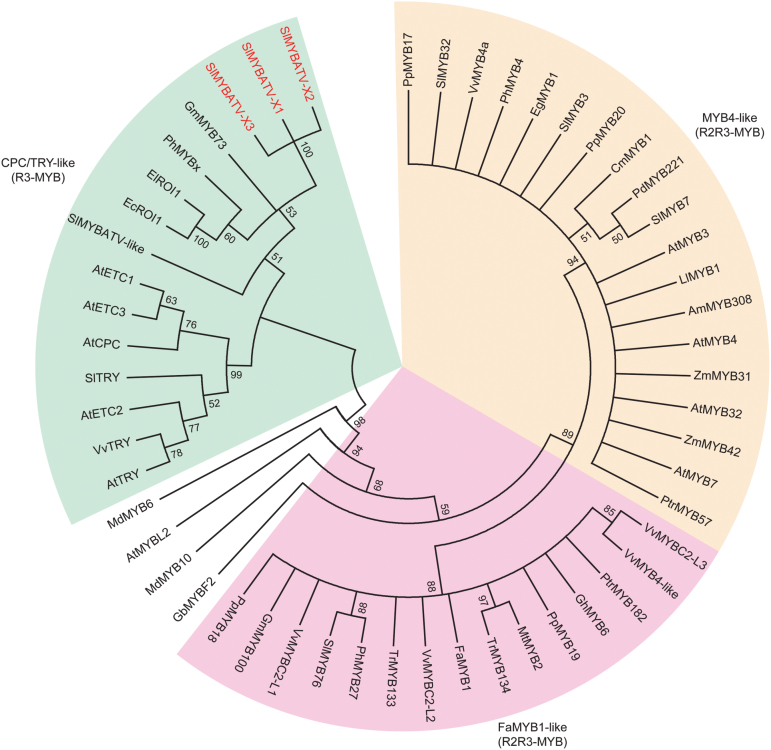
Phylogenetic analysis of SlMYBATV and the other MYB repressors. The phylogenetic tree was constructed by using MEGA6 (Tamura *et al.*, 2013) with 1000 bootstrap replicates, which was based on the N-terminal protein sequences comparing the R2R3 domain or only the R3 domain for the small MYB proteins. Numbers on the tree indicate the percentage of consensus support.

To identify the relationship between SlMYBATV, the candidate tomato MYB repressors, and the known MYB repressors for phenylpropanoid and flavonoid biosynthesis, multiple sequence alignments were performed (Supplementary Figs S3, S4), and a phylogenetic tree was constructed (Fig. 4). SlMYBATV was clustered with a group of R3 MYB TFs. Within this group, AtCPC, EcROI1 (*Erythranthe cardinalis* rose intensity 1), ElROI1 (*E. lewisii* rose intensity 1), and PhMYBx are negative regulators of anthocyanin biosynthesis (Kroon, 2004; Zhu *et al.*, 2009; Albert *et al.*, 2011, 2014; Yuan *et al.*, 2013; Nemie-Feyissa *et al.*, 2014). Within this group, SlMYBATV was more closely related to PhMYBx than AtCPC. Two putative tomato R3 MYB proteins, SlMYBATV-like and SlTRY, were also found within the same clade. SlMYBATV-like was more closely related to PhMYBx, and SlTRY was more closely related to AtCPC (Fig. 4). A study has suggested that SlTRY represses anthocyanin synthesis because the ectopic expression of *SlTRY* in Arabidopsis reduces anthocyanin accumulation (Nukumizu *et al.*, 2013). For the four putative tomato R2R3 MYB repressors, SlMYB3, SlMYB7, and SlMYB32 were clustered within the group of MYB4-like proteins, and SlMYB76 was clustered within a group of FaMYB1-like proteins and was closely related to PhMYB27, which is an anthocyanin repressor in petunia (Albert *et al.*, 2014; Jun *et al.*, 2015).

### Transcriptional analysis of structural genes related to anthocyanin biosynthesis

The anthocyanin-related R3 MYB repressors, such as AtCPC, EcROI1, ElROI1, and PhMYBx, repress anthocyanin biosynthesis through feedback inhibition of the expression of the structural genes and regulatory genes. *SlMYBATV*, the candidate gene of *ATV*, encodes a homolog of these R3 MYB repressors (Figs 2–4). In order to help us determine whether ATV plays a similar role in regulation of anthocyanin biosynthesis as these R3 MYB repressors, transcriptional expression analyses of anthocyanin structural and regulatory genes were performed using the fruit peel from an F_5_ population for *Aft*/*Aft ATV*/*ATV* (abbreviated as *Aft*/*Aft*) and *Aft*/*Aft atv*/*atv*. The two parents, Heinz1706 (*AFT*/*AFT ATV*/*ATV*) and Indigo Rose (*Aft*/*Aft atv*/*atv*), were used as controls.

**Fig. 5.**
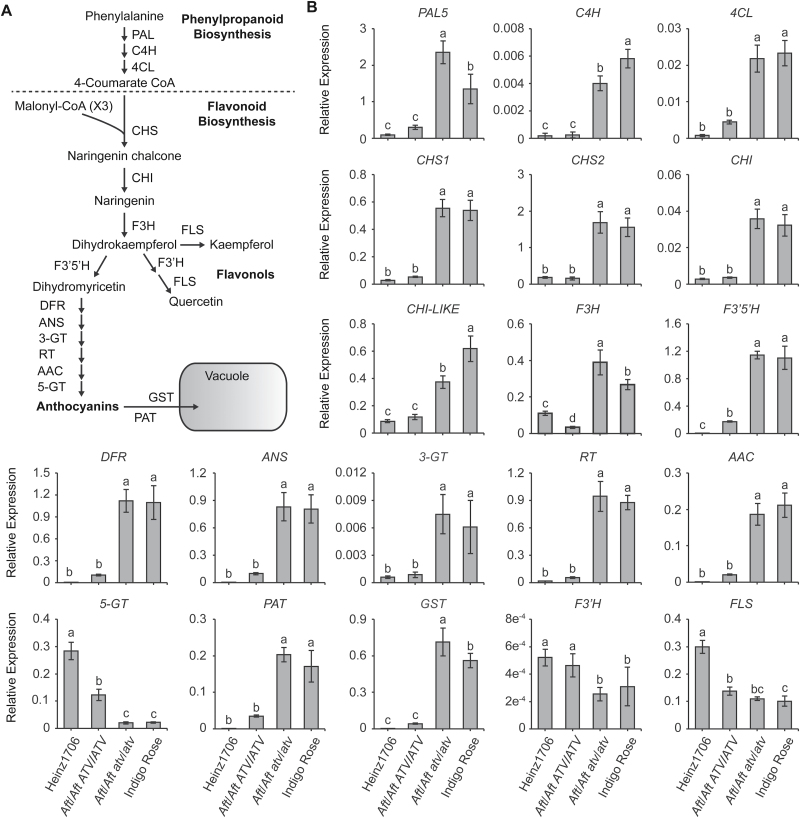
Transcriptional analysis of the structural genes of the anthocyanin biosynthetic pathway in the peel of tomato fruit at the mature green stage. (A) The anthocyanin biosynthetic pathway (modified from Povero *et al.*, 2011). (B) Analysis of the transcriptional expression of structural genes of the anthocyanin biosynthetic pathway was determined by qRT-PCR in the fruit peel of *Aft*/*Aft ATV*/*ATV* (abbreviated as *Aft*/*Aft*) and *Aft*/*Aft atv*/*atv* in a F_5_ population, and the two parent lines Heinz1706 (genotype, *AFT*/*AFT ATV*/*ATV*) and Indigo Rose (*Aft*/*Aft atv*/*atv*). A tomato *ACTIN* (*Solyc03g078400*) gene was used as the reference: relative expression levels are shown. Different letters indicate statistically significant differences among groups (Tukey’s honestly significant difference test, *P*<0.01). PAL, phenyl alanine ammonia-lyase; C4H, cinnamate-4-hydroxylase; 4CL, 4-coumaroyl:CoA-ligase; CHS, chalcone synthase; CHI, chalcone isomerase; CHI-LIKE, chalcone isomerase-like; F3H, flavanone 3-hydroxylase; F3′5′H, flavonoid 3′5′-hydroxylase; DFR, dihydroflavonol 4-reductase; ANS, leucoanthocyanidin dioxygenase; 3-GT, 3-O-glucosyltransferase; RT, rhamnosyl transferase; AAC, anthocyanin acyltransferase; 5-GT, 5-O-glucosyltransferase; PAT, putative anthocyanin transporter; GST, glutathione S-transferase; F3′H, flavonoid 3′-hydroxilase; FLS, flavonol synthase.

The *Aft*/*Aft atv*/*atv* fruit peel in the F_5_ population displayed more intense anthocyanin pigmentation than *Aft*/*Aft*, which was similar to that in the F_3_ population (Fig. 1). In the anthocyanin biosynthesis pathway, from phenylalanine to anthocyanins, all the structural genes that were detected in the present study were significantly up-regulated in the *Aft*/*Aft atv*/*atv* fruit peel at the mature green stage relative to *Aft*/*Aft*, except for gene *5-GT* (5-O-glucosyltransferase) (Fig. 5). At the fully ripened stage, all the structural genes were significantly up-regulated in *Aft*/*Aft atv*/*atv* relative to *Aft*/*Aft* (Supplementary Fig. S5). However, the specific flavonol-biosynthetic genes *F3′H* (flavonoid 3′-hydroxilase) and *FLS* (flavonol synthase) were not up-regulated in *Aft*/*Aft atv*/*atv* (Fig. 5 and Supplementary Fig. S5). The expression patterns of these structural genes in *Aft*/*Aft* and *Aft*/*Aft atv*/*atv* of the F_5_ population were also similar to those found previously in the fruit peel of tomato lines *Aft*/*Aft* and *Aft*/*Aft atv*/*atv* (Povero *et al.*, 2011), except for the *5-GT* gene. These results suggest that *ATV* encodes a negative regulator of expression of the structural genes and represses anthocyanin biosynthesis.

### Transcriptional analysis of the candidate anthocyanin regulatory genes of the MBW activation complex

The candidate anthocyanin regulatory genes of the MBW activation complex have been previously identified (Kiferle *et al.*, 2015). They include four MYB TFs, namely SlAN2 (Solyc10g086250) (De Jong *et al.*, 2004; Boches and Myers, 2007), SlANT1 (Solyc10g086260) (Mathews *et al.*, 2003; Schreiber *et al.*, 2012), SlANT1-like (Solyc10g086270) (Boches, 2009), and SlAN2-like (Solyc10g086290) (Boches, 2009); two bHLH TFs, namely SlAN1 (Solyc09g065100) and SlJAF13 (Solyc08g081140) (Kiferle *et al.*, 2015; Qiu *et al.*, 2016); and one WDR TF, SlAN11 (Solyc03g097340) (De Jong *et al.*, 2004; Kiferle *et al.*, 2015).

**Fig. 6.**
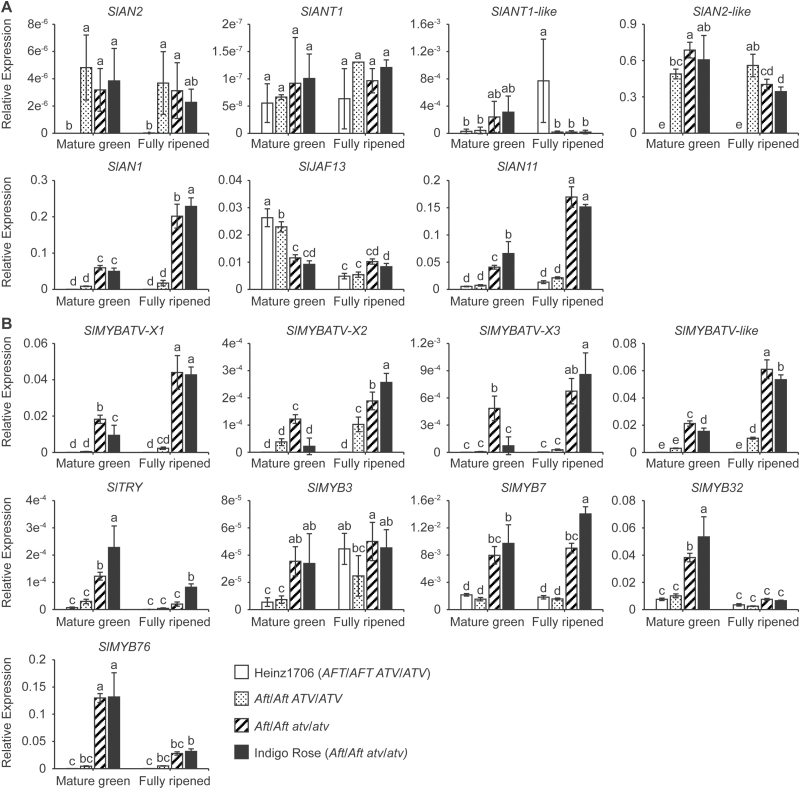
Transcriptional analysis of the candidate regulators of the anthocyanin biosynthetic pathway in the peel of tomato fruit. (A) The candidate members of the MBW activation complex. (B) The candidate MYB repressors of the anthocyanin biosynthetic pathway. A tomato *ACTIN* (*Solyc03g078400*) gene was used as reference: relative expression levels are shown. Different letters indicate statistically significant differences among groups (Tukey’s honestly significant difference test, *P*<0.01).

Two of the MYB TFs, *SlAN2* and *SlAN2*-like, were expressed at a much higher level in the fruit peel of the tomato lines harboring the *Aft* locus compared to that in the wild-type Heinz1706. In addition, the expression level of *SlAN2*-like was about 10 000-fold higher than that of *SlAN2* (Fig. 6A). The expression of *SlANT1* was barely detected in any of the tomato lines. At the mature-green stage, the wild-type *ATV* allele may have repressed the expression of *SlAN2*-like and *SlANT1*-like. Interestingly, at the fully ripened stage, *SlANT1*-like was expressed at a much higher level in the wild-type Heinz1706 compared to that of the lines harboring the *Aft* locus (Fig. 6A).

The expression patterns of the two bHLH TFs, *SlAN1* and *SlJAF13*, differed. The expression of *SlAN1* was up-regulated by *Aft* alone, and highly up-regulated in the *Aft* and *atv* double-mutants (Fig. 6A). However, *atv* might have repressed the expression of *SlJAF13* at the mature-green stage. The expression of WDR TF *SlAN11* might not be affected by *Aft*, but up-regulated by *atv*.

### Transcriptional analysis of the candidate MYB repressors for the phenylpropanoid and flavonoid pathways

The R3 MYB and R2R3 MYB repressors are also involved in the regulation of anthocyanin biosynthesis (Matsui *et al.*, 2008; Zhu *et al.*, 2009; Albert *et al.*, 2014; Nemie-Feyissa *et al.*, 2014). SlMYBATV, SlMYBATV-like, and SlTRY are putative R3 Myb repressors in tomato (Fig. 4 and Supplementary Figs S3, S4). Three kinds of transcripts of *SlMYBATV* (Fig. 2 and Supplementary Fig. S1) were all up-regulated in the *Aft*/*Aft atv*/*atv* fruit peel when compared to that in *Aft*/*Aft*; however, *SlMYBATV-X1* was expressed much higher than the other two (Fig. 6B). The expression pattern of *SlMYBATV*-*like* was similar to that of *SlMYBATV-X1*. *SlTRY* was also up-regulated in *Aft*/*Aft atv*/*atv* at the mature-green stage, although its expression level was much lower than that of *SlMYBATV-X1* and *SlMYBATV*-*like*.

SlMYB3, SlMYB7, SlMYB32, and SlMYB76 (Zhao *et al.*, 2014) were identified as the candidate R2R3 MYB repressors of the phenylpropanoid and flavonoid pathways in tomato (Fig. 4 and Supplementary Fig. S3). At the mature-green stage, all of the four *R2R3 MYB* genes were significantly up-regulated in the *Aft*/*Aft atv*/*atv* fruit peel when compared to *Aft*/*Aft*. However, at the fully ripened stage, the four *R2R3 MYB* genes were also up-regulated in *Aft*/*Aft atv*/*atv*, but only *SlMYB7* was significantly up-regulated (Fig. 6B).

## Discussion

### *SlMYBATV* is a candidate gene of the *atv* locus

Anthocyanins are potential health-promoting compounds in the human diet (He and Giusti, 2010; Li *et al.*, 2017), but unfortunately, none are present in cultivated tomato fruit (Gonzali *et al.*, 2009). The *atv*, *Aft*, and *Abg* loci from wild tomato species can promote anthocyanin pigmentation in fruit, and the *atv* locus can dramatically increase the amount of anthocyanins in cultivated tomato fruit when it is combined with either the *Aft* or *Abg* locus (Mes *et al.*, 2008). Transcriptional analysis of tomatoes with high anthocyanin content has suggested that ATV might be a repressor of anthocyanin biosynthesis (Povero *et al.*, 2011).

The R3 MYB repressors, such as AtCPC, EcROI1, ElROI1, and PhMYBx, play important roles in the feedback inhibition of anthocyanin biosynthesis. Loss-of-function mutations in these genes leads to higher expression levels of anthocyanin structural genes and stronger anthocyanin pigmentation (Kroon, 2004; Zhu *et al.*, 2009; Albert *et al.*, 2011, 2014; Yuan *et al.*, 2013; Nemie-Feyissa *et al.*, 2014). *SlMYBATV* was the only gene encoded in the *atv* locus region that was fine-mapped in this study (Fig. 2). Sequence alignment and phylogenetic analysis suggested that SlMYBATV was a homolog of AtCPC, EcROI1, ElROI1, and PhMYBx (Figs 3, 4 and Supplementary Figs S3, S4). A 4-bp insertion in the *atv* locus led to a truncated SlMYBATV protein (Fig. 3) that was related to higher expression levels of anthocyanin structural genes and stronger anthocyanin pigmentation in the fruit peel (Figs 1, 5 and Supplementary Fig. S5). Taken together, these findings suggest that *SlMYBATV* is a candidate gene of the *atv* locus and is a putative R3 MYB repressor of anthocyanin biosynthesis.

Molecular marker-assisted selection (MAS) may greatly increase the efficiency and effectiveness of breeding compared to conventional methods (Xu and Crouch, 2008). Anthocyanin-enriched tomato varieties have been developed by pyramiding of the *atv* and *Aft* loci (Mes *et al.*, 2008). However, *atv* is a recessive, and it is very difficult to distinguish plants containing the heterozygous *atv* locus (*ATV*/*atv*) from those containing the homozygous wild-type allele *ATV*/*ATV* through phenotypic evaluation (Fig. 1). Therefore, when it is introduced into elite tomato lines by using conventional phenotypic selection, additional selfing or test-crossing generations are required after every back-cross. These time-consuming steps could be eliminated in a MAS program, and homozygous and heterozygous back-cross plants could be distinguished with the aid of a co-dominant marker that is closely linked to the *atv* locus. In the present study, the *atv* locus was fine-mapped to a 5.0-kb region on chromosome 7 (Fig. 2). This region was flanked by two InDel markers: HP1917 was 16.9-kb upstream of this region and HP3217 was 9.6-kb downstream (Fig. 2, Supplementary Table S1). Another InDel marker, ATV-In, was developed according to the 4-bp insertion that led to a truncated SlMYBATV protein (Fig. 2 and Supplementary Fig. S2, Table S1). All these three InDel markers were co-dominant and could discriminate between the homozygous wild-type, heterozygous, and homozygous *atv* mutant genotypes. These markers may facilitate MAS of *atv* in breeding for anthocyanin-enriched tomato varieties.

### A putative model for the regulation of anthocyanin biosynthesis in the peel of tomato fruit

The biosynthesis of anthocyanins in plants is precisely regulated to adapt to various developmental and environmental signals. Based on findings for Eudicots (particularly Arabidopsis and petunia), a model has been built to describe the common features of the regulation network of anthocyanin biosynthesis (Albert *et al.*, 2014). In this model, biosynthesis is initiated by activating the expression of *R2R3 MYB* activator genes under inductive conditions. The R2R3 MYB activator interacts with bHLH1 (PhJAF13/AtEGL3 clade bHLH) and WDR to form a MBW activation complex that activates the expression of bHLH2 (PhAN1/AtTT8 clade bHLH). The R2R3 MYB activator, bHLH2, and WDR form a core MBW activation complex that activates the expression of bHLH2 and structural genes to promote anthocyanin accumulation. The core MBW activation complex also activates the expression of the *R2R3 MYB* and *R3 MYB* repressors genes. Feedback inhibition of anthocyanin biosynthesis is produced by the interaction of these MYB repressors with the core MBW activation complex (Albert *et al.*, 2014).

This model seems to be suitable for explaining the regulation of anthocyanin biosynthesis in the peel of tomato fruit, and tomato homologs of these regulators in this model seem to play similar roles. The candidate TFs in the anthocyanin-related MBW activation complexes in tomato have been identified. These include four MYB TFs (SlAN2, SlANT1, SlANT1-like, and SlAN2-like), two bHLH TFs (SlJAF13 and SlAN1), and one WDR TF, SlAN11 (Mathews *et al.*, 2003; Boches and Myers, 2007; Sapir *et al.*, 2008; Kiferle *et al.*, 2015; Qiu *et al.*, 2016). The four *MYB* TF genes belong to a MYB gene cluster that co-segregates with the *Aft* locus (Boches and Myers, 2007; Sapir *et al.*, 2008). The candidate tomato MYB repressors for the phenylpropanoid and flavonoid pathways were also identified in this study. These are three R3 MYB repressors (SlMYBATV, SlMYBATV-like, and SlTRY) and four R2R3 MYB repressors (SlMYB3, SlMYB7, SlMYB32, and SlMYB76) (Figs 3, 4 and Supplementary Figs S3 and S4). The fruit peel of the cultivated tomato Heinz1706 does not produce anthocyanin, but the peel of *Aft*/*Aft* presents purple spots due to anathocyanin accumulation (Fig. 1). *SlAN2*, *SlANT1*, *SlAN2-like*, *SlAN1*, *SlAN11*, *SlMYBATV*, *SlMYBATV-like*, *SlTRY*, *SlMYB76* and most of the anthocyanin structural genes were up-regulated in *Aft*/*Aft* fruit peel. The up-regulation of *SlAN2* and *SlAN2-like* was significant (Figs 5, 6 and Supplementary Fig. S5). *Aft*/*Aft atv*/*atv* fruit peel, containing the mutant allele of *SlMYBATV*, displayed stronger anthocyanin pigmentation than *Aft*/*Aft* (Fig. 1). *SlAN1*, *SlAN11*, *SlMYBATV*, *SlMYBATV-like*, *SlTRY*, *SlMYB7*, *SlMYB32*, *SlMYB76* and most of the anthocyanin structural genes were significantly up-regulated in *Aft*/*Aft atv*/*atv* fruit peel when compared to *Aft*/*Aft* (Figs 5, 6 and Supplementary Fig. S5). The relationship between anthocyanin content and transcript levels of these genes suggested that they might be involved in the regulation of anthocyanin biosynthesis in the peel of tomato fruit.

**Fig. 7.**
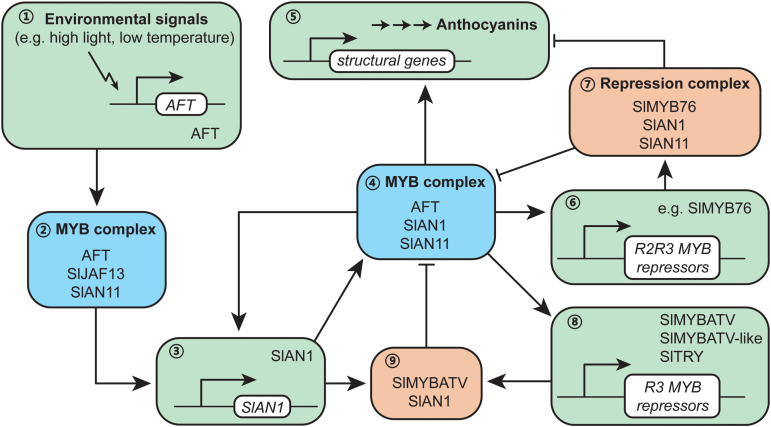
A putative model of the anthocyanin gene regulation network in the peel of tomato fruit. This model is modified from fig. 8 in the previously published paper [Albert *et al.* (2014). A conserved network of transcriptional activators and repressors regulates anthocyanin pigmentation in eudicots. The Plant Cell 26, 962–980. Republished with permission (www.plantcell.org), ‘Copyright American Society of Plant Biologists’] according to the expression patterns and putative functions of the corresponding genes that we determined in the present study. In the fruit peel of *Aft*/*Aft* (abbreviation of *Aft*/*Aft ATV*/*ATV*) plants (1) anthocyanin biosynthesis is initiated by activating the expression of the *AFT* gene under high light or low temperature. (2) The AFT protein (MYB activator, most likely SlAN2 or SlAN2-like) interacts with SlJAF13 and SlAN11 to form a MBW complex that activates the expression of SlAN1 (3). (4) SlAN1 interacts with AFT and SlAN11 to form a core MBW activation complex, and the complex activates the expression of the *SlAN1* gene and most of the structural genes (5), and enhances the anthocyanin pigmentation. (6) The MBW complex also activates the expression of R2R3 MYB repressors. (7) The R2R3 MYB repressor, most likely SlMYB76, might intrude into the MBW complex and repress the anthocyanin pigmentation. (8) The MBW complex also activates the expression of R3 MYB repressors. (9) The R2R3 MYB repressor, e.g. SlMYBATV, competes with AFT to bind SlAN1 to inhibit the formation of new MBW complexes. This feedback inhibition can prevent the production of too much anthocyanin.

A putative model for the regulation of anthocyanin biosynthesis in the peel of tomato fruit was constructed to explain the findings of the present study (Fig. 7), as follows.

(1) In *Aft*/*Aft* (abbreviation of *Aft*/*Aft ATV*/*ATV*) fruit peel, anthocyanin biosynthesis is initiated by activating the expression of the *AFT* gene (*R2R3 MYB* activator, most likely the *SlAN2* or *SlAN2-like* gene) under high light or low temperature (Povero *et al.*, 2011). The AFT protein interacts with SlJAF13 and SlAN11 to form a MBW activation complex that activates the expression of SlAN1. SlAN1, AFT, and SlAN11 form a core MBW activation complex that activates the expression of the *SlAN1* gene (Fig. 6A) and most of the anthocyanin structural genes (Fig. 5 and Supplementary Fig. S5) to promote the anthocyanin pigmentation (Fig. 1). The core MBW activation complex also activates the expression of the *R2R3 MYB* and *R3 MYB* repressors (Fig. 6B). These MYB repressors (e.g. SlMYBATV and SlMYB76) bind members of the core MBW activation complex to produce feedback inhibition of anthocyanin biosynthesis.

(2) In *Aft*/*Aft atv*/*atv* fruit peel, there is no feedback inhibition by SlMYBATV, because it loses the R3 MYB domain (Fig. 3) and cannot bind SlAN1 to inhibit the formation of new MBW activation complexes. Therefore, *Aft*/*Aft atv*/*atv* fruit peel presents higher expression levels of *SlAN1*, structural genes, and *MYB* repressor genes, and stronger anthocyanin pigmentation than *Aft*/*Aft* fruit peel (Figs 1, 5, 6).

(3) In *Aft*/*Aft ATV*/*atv* fruit peel, feedback inhibition by SlMYBATV still works, because the heterozygous *atv* locus can produce normal SlMYBATV. Therefore, the anthocyanin pigmentation of *Aft*/*Aft ATV*/*atv* fruit peel is similar to that of *Aft*/*Aft* (Fig. 1).

(4) In the fruit peel of cultivated lines (*AFT*/*AFT ATV*/*ATV*), the expression of the *AFT* gene (most likely *SlAN2* or *SlAN2-like*) is barely detected (Fig. 6A). So, there is no anthocyanin accumulation (Fig. 1) because its biosynthesis is not initiated. Due to the absence of the AFT protein, regardless of whether the SlMYBATV is normal or not, the feedback inhibition by SlMYBATV does not happen, because there is no MBW activation complex that promotes the expression of *SlMYBATV* and *SlAN1* (Figs 5, 6 and Supplementary Fig. S5). Therefore, the peel of *AFT*/*AFT atv*/*atv* fruit, similar to *AFT*/*AFT ATV*/*ATV*, also barely produces anthocyanin (Povero *et al.*, 2011).

In summary, in this study the *atv* locus was narrowed down to an approximately 5.0-kb interval on chromosome 7 (Fig. 2). *SlMYBATV*, a putative R3 MYB repressor, was identified as the candidate gene (Fig. 2). A 4-bp insertion in *SlMYBATV* transcripts from the *atv* locus resulted in a frame-shift and premature protein truncation (Fig. 3). A co-dominant InDel marker, ATV-In, developed according to the 4-bp insertion, can be used for marker-assisted selection of anthocyanin-enriched tomato cultivars, and *SlMYBATV* can also be used as the target for gene editing to quickly develop such cultivars. Several putative tomato MYB repressors were also identified in this study (Fig. 4). Anthocyanin pigmentation and the transcriptional expression of most of the structural genes, candidate regulatory activators, and regulatory repressors of anthocyanin biosynthesis could be promoted by the *Aft* locus, and this promotion could be enhanced when the *Aft* locus is combined with the *atv* locus (Figs 1, 5, 6). Based on the expression patterns and putative functions of the genes detected in this study we propose a model for the regulation of anthocyanin biosynthesis in the peel of tomato fruit (Fig. 7). In order to prove or modify this model, future work will focus on: the analysis of phenotypes of tomato lines with gain- and/or loss-of-function mutations of these regulator genes; the interactions between these regulator proteins; the interactions between these regulator proteins and the promoters of the target genes; and the transcriptional activation or repression activities of these regulator proteins.

## Supplementary data

Supplementary data are available at *JXB* online.

Fig. S1. Sequence polymorphism of the coding DNA sequence of *SlMYBATV*.

Fig. S2. Sequence polymorphism of the genomic sequence of *SlMYBATV*.

Fig. S3. Multiple sequence alignment of MYB flavonoid repressors based on their full-length proteins.

Fig. S4. Multiple sequence alignment of MYB flavonoid repressors based on the R3 and R2R3 domains.

Fig. S5. Transcriptional analysis of the structural genes of the anthocyanin biosynthetic pathway in the peel of tomato fruit at the fully ripened stage.

Table S1. General information on the DNA markers used in this study

Table S2. Primers used for sequencing and cDNA cloning.

Table S3. Information regarding the known MYB repressors for the phenylpropanoid and flavonoid pathways.

Table S4. Primers for quantitative RT-PCR.

Table S5. Preliminary mapping of the *atv* locus.

Supplementary Material
