# Supplementary material for: A putative R3 MYB repressor is the candidate gene underlying atroviolacium, a locus for anthocyanin pigmentation in tomato fruit
Source: J Exp Bot. 2017 Nov 25;68(21-22):5745–58. doi: 10.1093/jxb/erx382 (PMC5854135; doi:10.1093/jxb/erx382)
Supplement: Supplementary Material [file erx382_suppl_supplementary_material.pdf]

A putative *R3 MYB* repressor is the candidate gene underlying *atroviolacium*, a locus for anthocyanin pigmentation in tomato fruit

Xue Cao[1,†], Zhengkun Qiu[2,†], Xiaotian Wang[1], Tong Van Giang[1], Xiaolin Liu[1], Jing Wang[2], Xiaoxuan Wang[1], Jianchang Gao[1], Yanmei Guo[1], Yongchen Du[1], Guoping Wang[2], Zejun Huang[1,*]

[1] The Key Laboratory of Biology and Genetic Improvement of Horticultural Crops of the Ministry of Agriculture, Institute of Vegetables and Flowers, Chinese Academy of Agricultural Sciences, Beijing 100086, China

[2] Department of Vegetable Science, College of Horticulture, South China Agricultural University, Guangzhou 510642, China

[†] These authors contributed equally to this study

* Corresponding author, E-mail: huangzejun@caas.cn; Tel: 86-10-82109538

Supplementary data

A

```
SlMYBATV-X1  ATGGCAGATTGGAATAGATCAAGCACATCAGATAATGCCTCAGTGGTCTCACCTGAT----TCGACCCGAGTGGTT   72
slmybatv-x1  ATGGCAGATTGGAATAGATCAAGCACATCAGATAATGCCTCAGTGGTCTCACCTGATAGATTCGACCCGAGTGGTT   76

SlMYBATV-X1  GCATTAGAGACTACCAACGAAGAAACCTCTAAACTTGAATTTTCAGAAGATGAAGAAATGCTCATTGCTAAAATGT  148
slmybatv-x1  GCATTAGAGACTACCAACGAAGAAACCTCTAAACTTGAATTTTCAGAAGATGAAGAAATGCTCATTGCTAAAATGT  152

SlMYBATV-X1  TCAGCTTGGTTAGAGAGAGGTGGTCATTAATTGCTGGAAGAATCCCAGGAAGAAATGCTGATGAGATTGAAAAATA  224
slmybatv-x1  TCAGCTTGGTTAGAGAGAGGTGGTCATTAATTGCTGGAAGAATCCTAGGAAGAAATGCTGATGAGATTGAAAAATA  228

SlMYBATV-X1  TTGGAAATCAAAATACTCCAAAAGCCAGGAGGAAATACAGGCCCAATCTCAAGATGAAGCTCATGGCATTAGATTG  300
slmybatv-x1  TTGGAAATCAAAATACTCCAAAAGCCAGGAGGAAATACAGGCCCAATCTCAAGATGAAGCTCATGGCATTAGATTG  304

SlMYBATV-X1  ATAGAGAAAACTGGGCCTTCAACGGGGCACCCTCAGCAGTGTGTTTTTCCAGAAAAAAAGCCCATATCTGTCTGTC  376
slmybatv-x1  ATAGAGAAAACTGGGCCTTCAACGGCGCACCCTCAGCAGTGTGTTTTTCCAGAAAAAAAGCCCATATCTGTCTGTC  380

SlMYBATV-X1  AACCTTCAGATATTGGGCCTCAACTATTGGGCCCAACAACGAATGGGCCTCAACAGCTATTGGGCCCTAAAACAAC  452
slmybatv-x1  AACCTTCAGATACTGGGCCTCAACTATTGGGCCCAACAACGAATGGGCCTCAACAGCTATTGGGCCCTAAAACAAC  456

SlMYBATV-X1  TGGGCCCAATAACATCACTGTAGACCAAAAGTTAGTTAACCCAGCCCAAGACCAAATAAACCAATAA  519
slmybatv-x1  TGGGCCCAATAACATCACTGTAGACCAAAAGTTAGTTAACCCAGCCCAAGACCAAATAAACCAATAA  523
```

B

```
SlMYBATV-X2  ATGGCAGATTGGAATAGATCAAGCACATCAGATAATGCCTCAGTGGTCTCACCTGAT----TCGACCCGAGTGGTT   72
slmybatv-x2  ATGGCAGATTGGAATAGATCAAGCACATCAGATAATGCCTCAGTGGTCTCACCTGATAGATTCGACCCGAGTGGTT   76

SlMYBATV-X2  GCATTAGAGACTACCAACGAAGAAACCTCTAAACTTGAATTTTCAGAAGATGAAGAAATGCTCATTGCTAAAATGT  148
slmybatv-x2  GCATTAGAGACTACCAACGAAGAAACCTCTAAACTTGAATTTTCAGAAGATGAAGAAATGCTCATTGCTAAAATGT  152

SlMYBATV-X2  TCAGCTTGGTTAGAGAGAGGTGGTCATTAATTGCTGGAAGAATCCCAGGAAGAAATGCTGATGAGATTGAAAAATA  224
slmybatv-x2  TCAGCTTGGTTAGAGAGAGGTGGTCATTAATTGCTGGAAGAATCCTAGGAAGAAATGCTGATGAGATTGAAAAATA  228

SlMYBATV-X2  TTGGAAATCAAAATACTCCAAAAGCCAGATTTTGGGGTCAATGTAG  270
slmybatv-x2  TTGGAAATCAAAATACTCCAAAAGCCAGATTTTGGGGTCAATGTAG  274
```

C

```
SlMYBATV-X3  ATGGCAGATTGGAATAGATCAAGCACATCAGATAATGCCTCAGTGGTCTCACCTGAT----TCGACCCGAGTGGTT   72
slmybatv-x3  ATGGCAGATTGGAATAGATCAAGCACATCAGATAATGCCTCAGTGGTCTCACCTGATAGATTCGACCCGAGTGGTT   76

SlMYBATV-X3  GCATTAGAGACTACCAACGAAGAAACCTCTAAACTTGAATTTTCAGAAGATGAAGAAATGCTCATTGCTAAAATGT  148
slmybatv-x3  GCATTAGAGACTACCAACGAAGAAACCTCTAAACTTGAATTTTCAGAAGATGAAGAAATGCTCATTGCTAAAATGT  152

SlMYBATV-X3  TCAGCTTGGTTAGAGAGAGGTGGTCATTAATTGCTGGAAGAATCCCAGGAAGAAATGCTGATGAGATTGAAAAATA  224
slmybatv-x3  TCAGCTTGGTTAGAGAGAGGTGGTCATTAATTGCTGGAAGAATCCTAGGAAGAAATGCTGATGAGATTGAAAAATA  228

SlMYBATV-X3  TTGGAAATCAAAATACTCCAAAAGCCAGTAA  255
slmybatv-x3  TTGGAAATCAAAATACTCCAAAAGCCAGTAA  259
```

Fig. S1 Sequence polymorphism of the coding DNA sequence of *SlMYBATV*. Entire coding sequences were aligned by using Clustal X ver. 2 (Larkin *et al*., 2007) with default settings, and the conserved nucleotides were shaded by using GeneDoc (2.6) (Nicholas *et al*., 1997). *SlMYBATV-X1*, *SlMYBATV-X2*, and *SlMYBATV-X3* were three transcripts of the *SlMYBATV* gene from Heinz1706; *slmybatv-x1*, *slmybatv-x2*, and *slmybatv-x3* were three transcripts of the *SlMYBATV* gene from Indigo Rose.

```
Indigo-Rose   TGCCACCGTTTAATTAATTAAGGACATGTCCAATCTAGTTAATGTTGATTAAGAGTGTAAATACGGTTAAAAAGA     75
Heinz1706     TGCCACCGTTTAATTAATTAAGGACATGTCCAATCTAGTTAATGTTGATTAAGAGTGTAAATACGGTTAAAAAGA     75

Indigo-Rose   AAAAGAGATACTAATTATTTTTTGGGGAAAATATTTTGAATCATTAACACTAATAGAGGTAGTTTTAAACTAAAA    150
Heinz1706     AAAAGAGATACTAATTATTTTTTGGGGAAAATATTTTGAATCATTAACACTAATAGAGGTAGTTTTAAACTAAAA    150

Indigo-Rose   TATTAGTAATAGAGATATTTTTGGTATTTTGGTTCAATTTTACGTAGTTTAATTTTTTTTTAATTTCTTTCAGTA    225
Heinz1706     TATTAGTAATAGAGATATTTTTGGTATTTTGGTTCAATTTTACGTAGTTTAATTTTTTTTTAATTTCTTTCAGTA    225

Indigo-Rose   AAATATATAAATACTCCTATAGTTAACACTAGGATCGTATTAATAGTTTAATACCATACATAAATTAATATTAGG    300
Heinz1706     AAATATATAAATACTCCTATAGTTAACACTAGGATCGTATTAATAGTTTAATACCATACATAAATTAATATTAGG    300

Indigo-Rose   AATTTATAGATGTGAATAGCGAATAATAACTCATTATACATAGAGATATAATAATGTAAGACAATTATCTCTTAT    375
Heinz1706     AATTTATAGATGTGAATAGCGAATAATAACTCATTATACATAGAGATATAATAATGTAAGACAATTATCTCTTAT    375

Indigo-Rose   GATACAATTTTTTTTCTTACTAAATTTCACATATAGTAAAAAACAAGACGAAATTTATACCTTTGTAAAATCCAT    450
Heinz1706     GATACAATTTTTTTTCTTACTAAATTTCACATATAGTAAAAAACAAGACGAAATTTATACCTTTGTAAAATCCAT    450

Indigo-Rose   CTTGTCCAATTATGTTGAAGTGTATAGACAATTTTAATGAGGAAGCAAATCAAGAAAAATGAGCTTCCTCTAGTA    525
Heinz1706     CTTGTCCAATTATGTTGAAGTGTATAGACAATTTTAATGAGGAAGCAAATCAAGAAAAATGAGCTTCCTCTAGTA    525

Indigo-Rose   CAGATTTTCAAAGGTTCACAAATGTGATTTAACACTAATAAAGCCAAGATGAATTACCTTAAACAAAGGGCATAT    600
Heinz1706     CAGATTTTCAAAGGTTCACAAATGTGATTTAACACTAATAAAGCCAAGATGAATTACCTTAAACAAAGGGCATAT    600

Indigo-Rose   GTATGTCTGTTAACTAGCAATTCGTCCTTCCGTTTCATATCAGTTTTCATATTTATTTTTTAAAAAATTAATTTA    675
Heinz1706     GTATGTCTGTTAACTAGCAATTCGTCCTTCCGTTTCATATCAGTTTTCATATTTATTTTTTAAAAAATTAATTTA    675

Indigo-Rose   ACTAATTCTTTTCCATAATTTAAAATTCAATTGATACAAAAATTATTATAAATTATAATTTTTTTCTCAAGTCAA    750
Heinz1706     ACTAATTCTTTTCCATAATTTAAAATTCAATTGATACAAAAATTATTATAAATTATAATTTTTTTCTCAAGTCAA    750

Indigo-Rose   CATTAATAACAATATGTTCAGAGGAATCCCGCAAATGAAATATAGAGAAGATAAAGTGTATGTAGTACTTGTCAT    825
Heinz1706     CATTAATAACAATATGTTCAGAGGAATCCCGCAAATGAAATATAGAGAAGATAAAGTGTATGTAGTACTTGTCAT    825

Indigo-Rose   GATTCGTAGAAGTAGATAAATTGTTTCTGAAAAAACATTGCTTCAACATTCAAGAATAAGAAAACATAGCAACTA    900
Heinz1706     GATTCGTAGAAGTAGATAAATTGTTTCTGAAAAAACATTGCTTCAACATTCAAGAATAAGAAAACATAGCAACTA    900

Indigo-Rose   AAAAAGGAAGTAGTGGAAATCCTACAAGAAAGGAACAAAAACAACAAAATAGTGTGATAATTAAAATACAGTAAA    975
Heinz1706     AAAAAGGAAGTAGTGGAAATCCTACAAGAAAGGAACAAAAACAACAAAATAGTGTGATAATTAAAATACAGTAAA    975

Indigo-Rose   CAATTAAAGATAATAGATATGATCCAAAGAAAGAACTATAGTACAATGACAAACACCAATGAGCCCAAACCCTCA   1050
Heinz1706     CAATTAAAGATAATAGATATGATCCAAAGAAAGAACTATAGTACAATGACAAACACCAATGAGCCCAAACCCTCA   1050

Indigo-Rose   AAAATAAGAAAACACTCCACTGTCTATTATAATAGACGTCCTTCACACCTTTATATATAAAATTATCTATCCTCG   1125
Heinz1706     AAAATAAGAAAACACTCCACTGTTTATTATAATAGACGTCCTTCATACCTTTATATATAAAATTATCTATCCTCG   1125

Indigo-Rose   CGGATAACCTGAAGTTTTGCAATATCCAGTCTGATCACTTTTTCTCAATACTTCTTTGCCTACATCTAAATCTTC   1200
Heinz1706     CGGATAACCTGAAGTTTTGCAATATCCAGTCTGATCACTTTTTCTCAATACTTCTTTGCCTACATCTAAATCTTC   1200

Indigo-Rose   TAGTACAATACCATAATCAACTTCTTGCACCTGCTCCCTGGAGCGTCTGTACATCTCTTCTTTACAAAGGTATAA   1275
Heinz1706     TAGTACAATACCATAATCAACTTCTTGCACCTGCTCCCTGGAGCGTCTGTACATCTCTTCTTTACAAAGGTATAA   1275

Indigo-Rose   ATTTGTAAAGGTATAAATTTTCTAAACATCCTCATCGCCTCAACATTCTCATTGACTCAACATGTATATCTGAAC   1350
Heinz1706     ATTTGTAAAGGTATAAATTTTCTAAACATCCTCATCGCCTCAACATTCTCATTGACTCAACATGTATATCTGAAC   1350

Indigo-Rose   ATGAAAGTTCTTGACAAGCTAACATTGCGCTTGATACAACAAAATCTATCTAACTATCACTTAATAAAAAACTTA   1425
Heinz1706     ATGAAAGTTCTTGACAAGCTAACATTGCGCTTGATACAACAAAATCTATCTAACTATCACTTAATAAAAAACTTA   1425

Indigo-Rose   CCTTCAA-GTTTCAATGGTACATTTTATCAAGTAAGACTCCGGATGCAACCTTCCACTTCATTCACGTACAGTCG   1499
Heinz1706     CCTTCAAAGTTTCAATGGTACATTTTATCAAGTAAGACTCCGGATGCAACCTTCCACTTCATTCACGTACAGTCG   1500
```

```
Indigo-Rose   TGCACCAATACGATGTGTGATATCATCGTCAATATCTCCATTTTCCTCGATTGAAAATCCAATTTACTTGAAACT   1574
Heinz1706     TGCACCAATACGATGTGTGATATCATCGTCAATATCTCCATTTTCCTCGATTGAAAATCCAATATACTTGAAACT   1575

Indigo-Rose   ATAATGAAAAAATATTTTGAAATCATGTTTATCAAACATACATAGTTTCACTCTCAAAAAATAAAACATGACATA   1649
Heinz1706     ATAATGAAAAAATATTTTGAAATCATGTTTATCAAACATACATAGTTTCACTCTCAAAAAATAAAACATGACATA   1650

Indigo-Rose   CTAATTTGAAACAAAAGAAGCATTTGCTTAGCACGATTAATAATTAAAATAAAAATACTTCTAGTATTGAGGAGT   1724
Heinz1706     CTAATTTGAAACAAAAGAAGCATTTGCTTAGCACGATTAATAATTAAAATAAAAATACTTCTAGTATTGAGGAGT   1725

Indigo-Rose   AGTAACACTTCGTTGTTTGGTAAAAATGTTGAAAATAATTTCCAACTAACCAATCGATATTTGAGTATCCACTTA   1799
Heinz1706     AGTAACACTTCGTTGTTTGGTAAAAATGTTGAAAATAATTTCCAACTAACCAATCGATATTTGAGTATCCACTTA   1800

Indigo-Rose   CTTCTTTGGCCTACTTATACCTCTCCTCGTACATACCATAACTAACTTCTCGCACCTCTTCACTGGGGCATCTGC   1874
Heinz1706     CTTCTTTGGCCTACTTATACCTCTCCTCGTACATACCATAACTAACTTCTCGCACCTCTTCACTGGGGCATCTGC   1875

Indigo-Rose   GCATATTCTCTTCACATGTTCAAATCATTTTAATCTTAATTCTATCATCTTGTCCACCACGGACTACGGAGCCAC   1949
Heinz1706     GCATATTCTCTTCACATGTTCAAATCATTTTAATCTTAATTCTATCATCTTGTCCACCACGGACTACGGAGCCAC   1950

Indigo-Rose   TCTCATCTTATTTAGAATATTCTCAACATCCTCATTAACTCGACATGCATCTTCTGAACATGGGTAGCCATCACT   2024
Heinz1706     TCTCATCTTATTTAGAATATTCTCAACATCCTCATTAACTCGACATGCATCTTCTGAACATGAGTAGCCATCACT   2025

Indigo-Rose   CCGCCTTATACAACAAAGTTTGTCTAACTACCAACTTATAGAAACTTACCTTGAAATTTCAATGGTACATTTTTG   2099
Heinz1706     CCGCCTTATACAACAAAGTTTGTCTAACTACCAACTTATAGAAACTTACCTTGAAATTTCAATGGTACATTTTTG   2100

Indigo-Rose   TCATACAAGACTCCGGATGCAACCTTTCACTTCATTCACATGCACCAATACGATATATAATATCATCGTCAATAT   2174
Heinz1706     TCATACAAGACTCCGGATGCAACCTTTCACTTCATTCACATGCACCAATACGATATATAATATCATCGTCAATAT   2175

Indigo-Rose   CTCCATTTCCTTCTATTATAAATTCAAGATACTTGAAACTATGATAAAAAAAAATACTTCAAAATCGTATTCACC   2249
Heinz1706     CTCCATTTCCTTCTATTATAAATTCAAGATACTTGAAACTATGATAAAAAAAA-TACTTCAAAATCGTATTCACC   2249

Indigo-Rose   AAAATTTCACAGTTTCACTCTCGAAAAATAAAACATGACACGAAAGAAGAATTTGTATCGTACAATTCATAATTA   2324
Heinz1706     AAAATTTCACAGTTTCACTCTCGAAAAATAAAACATGACACGAAAGAAGAATTTGTATCGTACAATTCATAATTA   2324

Indigo-Rose   AAATAAAAATAGTTCTAATATTGAGGAGTAGTAACACTTCGTTGTTTGGTGAAAATGTTAGGCAAATAATTTCCA   2399
Heinz1706     AAATAAAAATAGTTCTAATATTGAGGAGTAGTAACACTTCGTTGTTTGGTGAAAATGTTAGGCAAATAATTTCCA   2399

Indigo-Rose   ACTAACCAGTCGATATTTGAGTATCCACCTACCTCAATTTATTGGACCACCACACACTTCACTGTCATCTACCAT   2474
Heinz1706     ACTAACCAGTCGATATTTGAGTATCCACCTACCTCAATTTATTGGACCACCACACACTTCACTGTCATCTACCAT   2474

Indigo-Rose   TCTTCATATGTTTGTCTTTTTTTTATTTTATTCACTCAAATCAGAACCACCCCTTCTTTTTAGAATCCTTTTTTT   2549
Heinz1706     TCTTCATATGTTTGTCTTTTTTTTATTTTATTCACTCAAATCAGAACCACCCCTTCTTTTTAGAATCCTTTTTTT   2549

Indigo-Rose   TTTAATCATATAGTAGTAATAAAATTTTCTATGTATATATATAGACCACATTCAAATCTTTGTTCTCCACATATA   2624
Heinz1706     TTTAATCATATAGTAGTAATAAAATTTTCTATGTATATATATAGACCACATTCAAATCTTTGTTCTCCACATATA   2624
```

Start code

```
Indigo-Rose   CTCTTCCTTAATTAGTACTCCAATTCATTTAATTAAAGTTTTATTTCTCTTGATGGCAGATTGGAATAGATCAAG   2699
Heinz1706     CTCTTCCTTAATTAGTACTCCAATTCATTTAATTAAAGTTTTATTTCTCTTGATGGCAGATTGGAATAGATCAAG   2699

Indigo-Rose   CACATCAGATAATGCCTCAGTGGTCTCACCTGGTAATTAACAATTCTTTTTTATTTTTATCGCAAGAGCTTTTAA   2774
Heinz1706     CACATCAGATAATGCCTCAGTGGTCTCACCTGGTAATTAACAATTCTTTTTTATTTT-ATCGCAAGAGCTTTTAA   2773

Indigo-Rose   TTATTCTTTTCGTTCACTTTTATTGATCTATTTTGGACATCTCATGCTCCTTACCAAAATTTTTTTTGATATAA   2849
Heinz1706     TTATTCTTTTCGTTCACTTTTATTGATCTATTTTGGACATCTCATGCTCCTTACCAAAAATTTCTTTTGATATAA   2848

Indigo-Rose   GGGTCCTACAGTACATATTAATTGATATTTGATATTATGTTTTGAAAGAAGATTTGAGAAATAAATAACTAATGA   2924
Heinz1706     GGGTCCTACAGTACATATTAATTGATATTTGATATTATGTTTTGAAAGAAGATTTGAGAAATAAATAACTAATGA   2923

Indigo-Rose   GCTAAGGGTAAACATGAAATAAAATATTTGTCTTTTTTTCATGTTAAAAAGTAACGAGTAAAAATGAACGGATAC   2999
Heinz1706     GCTAAGGGTAAACATGAAATAAAATATTTGTCTTTTTTTCATGTTAAAAAGTAACGAGTAAAAATGAACGGATAC   2998
```

```
Indigo-Rose  TTTTATATTTGCTTATAAATATATTCCTTAAAGGAATTTGGAGAACATTATGATATGATTATCTGCGTCTAACCA  3074
Heinz1706    TTTTATATTTGCTTATAAATATATTCCTTAAAGGAATTTGGAGAACATTATGATATGATTATCTGCGTCTAACCA  3073

Indigo-Rose  TATACTCTAATAATGCCATAAGTAAACAAATATTTATCCTTTTGGCTACTTCCAAAATACATGTTCATTTATGAA  3149
Heinz1706    TATACTCTAATAATGCCATAAGTAAACAAATATTTATCCTTTTGGCTACTTCCAAAATACATGTTCATTTATGAA  3148

Indigo-Rose  ATCATTTTTTTTAATAATAAGTTAGTTAGTCGGAATTTAGACTTTAAAATTTATATATTTTTTTACATCAAGTTA  3224
Heinz1706    ATCATTTTTTTTAATAATAAGTTAGTTAGTCGGAATTTAGAATTTAAAATTTATGTATTTTTATACATCAAGTTA  3223

Indigo-Rose  ATATATTATACTACTTATAAGTTCACAATTAAATATTCAATTTTGTTAATAATTTTCTTAATATATTTATAAGTC  3299
Heinz1706    ATATATTACACTACTTATAAGTTCACAATTAAATATTCAATTTTGTTAATAATTTTCTTAATATATTTATAAGTC  3298
```

4-bp insertion

```
Indigo-Rose  TAAATAAAAGTTATTGAGTTCACGTGAATTCATTATAGATAGATTCGACCCGAGTGGTTGCATTAGAGACTACCA  3374
Heinz1706    TAAATAAAAGTTATTGAGTTCACGTGAATTCATTATAGA----TTCGACCCGAGTGGTTGCATTAGAGACTACCA  3369

Indigo-Rose  ACGAAGAAACCTCTAAACTTGAATTTTCAGAAGATGAAGAAATGCTCATTGCTAAAATGTTCAGCTTGGTTAGAG  3449
Heinz1706    ACGAAGAAACCTCTAAACTTGAATTTTCAGAAGATGAAGAAATGCTCATTGCTAAAATGTTCAGCTTGGTTAGAG  3444

Indigo-Rose  AGAGGTACGTTTAATATTTTTTTAAAAAAATTCTTGAATTTTGTGTATTAATTATTTAAGGTTTAATATAAAATT  3524
Heinz1706    AGAGGTACGTTTAATATTTTTTTAAAAAAATTCTTGAATTTTGTGTATTAATTATTTAAGGTTTAATATAAAATT  3519

Indigo-Rose  GTCATGGTGTAATTTTAATTAGGTGGTCATTAATTGCTGGAAGAATCCTAGGAAGAAATGCTGATGAGATTGAAA  3599
Heinz1706    GTCATGGTGTAATTTTAATTAGGTGGTCATTAATTGCTGGAAGAATCCCAGGAAGAAATGCTGATGAGATTGAAA  3594

Indigo-Rose  AATATTGGAAATCAAAATACTCCAAAAGCCAGTAAATTTCTTCAAATTACTTTTTCCCTCAGATGAATATTTTTA  3674
Heinz1706    AATATTGGAAATCAAAATACTCCAAAAGCCAGTAAATTTCTTCAAATTACTTTTTCCCTCAGATGAATATTTTTA  3669

Indigo-Rose  TCTATTTTTATTTATCTAATATTAATTCGATGTACTTCATAATATTTTTAAAAATTTCATAGTAAATAACAAAGA  3749
Heinz1706    TCTATTTTTACTTATCTAATATTAATTCGATGTACTTCATAATATTTTTAAAAATTTCATAGTAAATAACAAAGA  3744

Indigo-Rose  TAAATAATAAGTGATAAGCTTATCTCTAGATTTCTAAGTTGGATAAGTAAGAATGTTGTAGAAACTAGAGAGGAA  3824
Heinz1706    TAAATAATAAGTGATAAGCTTATCTCTAGATTTCTAAGTTGGATAAGTAAGAATGTTGTAGAAACTAGATAGGAA  3819

Indigo-Rose  GTAGTTCTTTCGTTCACTTCTATTTATCTAACTTATCTACTGTAGATTATAATTTTTTAAAAAATTACTTTTAAA  3899
Heinz1706    GTAGTTCTTTCGTTCACTTCTATTTATCTAACTTATCTACTGTAGATTATAATTTTTTTTAAAAATACTTTTAAA  3894

Indigo-Rose  ACTTATTTCTCAAATTATTTTTCAAGACTTAGAGATTAAAATCAAATATATATATATTTAACATAGGGTAGAGGA  3974
Heinz1706    ACTTATTTTTCAAATTATTTTTCAAGACTTAGAGATTAAAATCAA---TATATATATTTAACATAGGGTAGAGGA  3966

Indigo-Rose  CAAAAATTACATACTTTTAAGGTAAAATTACTATTTGTTCCTTGTAAATTTATAATTGCAAATATCTCTCAAACT  4049
Heinz1706    CAAAAATCACATACTTTTAAGGTAAAATTACTATTTGTTCCTTGTAAATTTATAATTACAAATATCTCTCAAACT  4041

Indigo-Rose  GATACAATAATACAAACGTTGATACATTAATCTAATTGCGCGGATACATTATTCGTTAGGTAAGATACAATAAAT  4124
Heinz1706    GATACAATAATACAA----------------------GCGCGAATACATTATTCGTTAGGTAAGATACAATAAAT  4094

Indigo-Rose  TTTATATATGATACACTGATCTAATGTATATTTTATACATGATACACTAATCTGATGCGCGGAATACATTAATAT  4199
Heinz1706    TTTATATA-----CACTGATCTAATGTATATTTTATACATGATACACTAATCTGATGCGCAAGATACATTAATAT  4164

Indigo-Rose  GAAGTGTGAAAATGAAAATTTTGGAAATTTATAAAACTAATAGGAAATAATTATAACAAAAATTTAAAAATGAAA  4274
Heinz1706    GAAGCGTGAAAATG-------------------------------------------------------------  4178

Indigo-Rose  TTTTTGTCATATATCCTTTAATATAAGTATTGTAATAAAATACTCATGTGAAACATTATTTTTTAAAAGAATATA  4349
Heinz1706    ---------------------------------------------------------------------------     -

Indigo-Rose  TAAAGTCTAATTAAACAAATAAGAATGAACAAAGAAAGTATATCATCACTTCAACCTAGCTGAATGCAATGACAG  4424
Heinz1706    ---------------------------------------------------------------------------     -

Indigo-Rose  TACAAGCTAAAATATGTTTTTGAACAAGATTTCAATTTTAGGAATTTGAATTTTTCAAAAAATTATATAGTATCG  4499
Heinz1706    ---------------------------------------------------------------------------     -
```

```
Indigo-Rose  TAGATATTAAATATATTATTTATAAAATATTTTCAAAATAAAAATATAAAGTTTACCTAAGTCTAAGATGATTAA  4574
Heinz1706    ---------------------------------------------------------------------------     -

Indigo-Rose  ATTCTGTTAAACTCGTAATCGTAAACAAACTTGAAACTCCGTCCCTTGTGGTTTATGTACTTTCAGAAGTTCGTG  4649
Heinz1706    ------TTAAATTCGTAATCGTAAACAAACTTGAAACTCCGTCCCTTGTGGTTTATGTACTTTCAGAAGTTCGTG  4247

Indigo-Rose  TATCTTGCATGAAATTTAATATTTTTATTATTTGAACAGTATACACAGTGAATATCATGTCATTTCAGGATCTTC  4724
Heinz1706    TATCTTGCATGAAATTTAATATTTTTATTATTTGAACAGTATACACAGTGAATATCATGTCATTTCAGGATCTTC  4322

Indigo-Rose  ACATTTACTTTATTTTTACTTTATCTACTTTTAATTTAACGATCCTTAGAAAAAATGATATAATTATTTTATGAT  4799
Heinz1706    ACATTTACTTTATTTTTACTTTATCTACTTTTAATTTAACGATCCTTAGAAAAAATGATATAATTATTTTATGAT  4397

Indigo-Rose  AATATTTGTATTAATTGATGTTTAATATTATCTTTTGAAAAATAATTTAGAAAATAAGTAATTAATGTTAAGAAA  4874
Heinz1706    AATATTTGTATTAATTGATGTTTAATATTATCTTTTGAAAAATAATTTAGAAAATAAGTAATTAATGTTAAGAAA  4472

Indigo-Rose  AAAATCAAATATTTTTATACGTTAAAAGTGACAAGTAAATGCAAATAAAAAGTATTATTCACTAGATATCAATAA  4949
Heinz1706    AAAATCAAATATTTTTATACGTTAAAAGTGACAAGTAAATGCAAATAAAAAGTATTATTCACTAGATATCAATAA  4547

Indigo-Rose  GCGAGTAAAAATATTCAGTAAGAAGATTCAACATATATTATATATATGAACATAAAAAAAAATTTCAATCCTTTT  5024
Heinz1706    GCGAGTAAAAATATTCAGTAAGAAGATTCAACATATATTATATATATGAACATAAAAAAAAATTTCAATCCTTTT  4622

Indigo-Rose  TGATTCTTTGGTGACTTGAACTTACATCCTTAGTATAATGATTGTTTACCATTTGAGCAACTTCCATGAAAATCT  5099
Heinz1706    TGATTCTTTGGTGACTTAAACTTACATCCTTAGTATATTGATTGTTTACCATTCGAGCAACTTCCATGAAAATCT  4697

Indigo-Rose  TTTCATTATCTGTAACGTTTTTATTAGGAGTTTTTTTAAAAAAAAAAATAATAAAAACAAAAATTTGTACTCTTC  5174
Heinz1706    TTTCATTATCTGTAACGTTTTTATTAGGAGTTTTTTTAAAAAAAATAATAATAAAAACAAAAATTTGTACTCTTC  4772

Indigo-Rose  ATTTAGGGAGGAAATACAGGCCCAATCTCAAGATGAAGCTCATGGCATTAGATTGATAGAGAAAACTGGGCCTTC  5249
Heinz1706    ATTTAGGGAGGAAATACAGGCCCAATCTCAAGATGAAGCTCATGGCATTAGATTGATAGAGAAAACTGGGCCTTC  4847

Indigo-Rose  AACGGCGCACCCTCAGCAGTGTGTTTTTCCAGAAAAAAAGCCCATATCTGTCTGTCAACCTTCAGATACTGGGCC  5324
Heinz1706    AACGGGGCACCCTCAGCAGTGTGTTTTTCCAGAAAAAAAGCCCATATCTGTCTGTCAACCTTCAGATATTGGGCC  4922

Indigo-Rose  TCAACTATTGGGCCCAACAACGAATGGGCCTCAACAGCTATTGGGCCCTAAAACAACTGGGCCCAATAACATCAC  5399
Heinz1706    TCAACTATTGGGCCCAACAACGAATGGGCCTCAACAGCTATTGGGCCCTAAAACAACTGGGCCCAATAACATCAC  4997

Indigo-Rose  TGTAGACCAAAAGTTAGTTAACCCAGCCCAAGACCAAATAAACCAATAACTTATGCCCAGCCCAGCCCAAGATTG  5474
Heinz1706    TGTAGACCAAAAGTTAGTTAACCCAGCCCAAGACCAAATAAACCAATAACCTATGCCCAGCCCAGCCCAAGATTG  5072

Indigo-Rose  GTCAAACATGACACTAGACAAACCTACAATATATTTTCTATTTACATATTTTCGCAAATACCTACTACTCAATTA  5549
Heinz1706    GTCAAACATGACACTAGACAAACCTACAATATATTTTCTATTTACATATTTTCGCAAATACCTACTACTCAATTA  5147

Indigo-Rose  TGCTATGTATAGCTATAGTTTGCATATTTACTAGTTGTAGTTACAGTTTAAGTTATCTCGTTTGTATAATTTACA  5624
Heinz1706    TGCTATGTATAGCTATAGTTTGCATATTTACTAGTTGTAGTTACAGTTTAAGTTATCTCGTTTGTATAATTTCCA  5222

Indigo-Rose  TGTATGTTTAAATAAGTGAGTTATATTTGTATAAACTCAAAATAATGAATTTATACAAACACAAACATTTAAACT  5699
Heinz1706    TATATGTTTAAATAAGTGAGTTATATTTGTATAAACTCAAAATAATGAATTTATACAAACACAAACATTTAAACT  5297

Indigo-Rose  TTAAATAGTTGTACAGATATATTATACAAAAGTTATACAAATTATATTATACAACTTTCGAATTATACACACTCG  5774
Heinz1706    TTAAATAGTTGTACAGATATATTATACAAAAGTTATACAAATTATATTATACAACTTTCCAATTATACACACTCG  5372

Indigo-Rose  ATTTGATTTTCTTGGAAGTGTTTGACGACACAATTGTTTATATAGACCATGATCATGATCATAATATTTGTGTAT  5849
Heinz1706    ATTTGATTTTCTTGGAAGTGTTTGACGACACAATTGTTTATATAGACCATGATCATGATCATAATATTTGTGTAT  5447

Indigo-Rose  TTGATTAAACTTATAATGTTATTAAATTAATTTATACGTACTTTTTAATAAGTTACAGATATCTCATATCCTTTC  5924
Heinz1706    TTGATTAAACTTATAATGTTATTAAATTAATTTATACGTACTTTTTAATAAGTTACAGATATCTCATATCCTTTC  5522

Indigo-Rose  TAATAAAATATCTTATATCCTTTCTAATAACCTCTCCTCGAACAATTGCTTTAAGTTTCAAAAATTGACTATTTC  5999
Heinz1706    TAATAAAATATCTTATATCCTTTCTAATAACCTCTCCTCGAACAATTGCTTTAAGTTTCAAAAATTGACTATTTC  5597

Indigo-Rose  CTATTTCATTTTTTGATAGTTCCCTATTAAGGGAAAATTACGTGGAATGACAAACATTCCTAAGTAATTAGTCAA  6074
Heinz1706    CTATTTCATTTTTTGATAATTCC-TATTAAGGGAAAATTACGTGGAATGACAAACATTCCTAAGTAATTAGTCAA  5671
```

```
Indigo-Rose   TAAGGGTATATTTTAATTTATTTACTCAATAACTATAGTTTCATTTTTTTGCCATAATTAATGGGACCCACCATC   6149
Heinz1706     TAAGGGTATATTTTAATTTATTGACTCAATAACTATAGTTTCATTTTTTTGCCATAATTAATGGGACCCACCATC   5746

Indigo-Rose   TCTTTGTATATCCAACCATGAAACAAAAAGTTATCTTTTTATATATAATTTTATCAAATCAATTTTATCTCTCCT   6224
Heinz1706     TCTTTGTATATCCAACCATGAAACAAAAAGTTATCTTTTTATATATAATTTTATCAAATCAATTTTATCTCTCCT   5821

Indigo-Rose   ACACTCATTCTATATCTTTCTTCTAAAATCACTCCTCCACTCCTATTTTGAATTTTGAATTTTGGAAAATTTCAC   6299
Heinz1706     ACACTCATTCTATATCTTTCTTCTAAAATCACTCCTCCACTCCTATTTTGAATTTTGAATTTTGGAAAATTTCAC   5896

Indigo-Rose   CATATCCCTGTTGTCTTACTTTCATTAATCCAATTTTTTTTAAAAAAATTTCTTCATCAACACAATTATATATGT   6374
Heinz1706     CATATCCCTGTTGTCTTACTTTCATTAATCCAATTTTTTTTTAAAAAAATTTCTTCATCAACACAATTATATATGT   5971

Indigo-Rose   ATTATTTTTCTTAGTTTTTTTCATATTTTTTTTAAAAAAAATTCTTTCTTTCTTTTCAGTTTAAAGTTCTTCAAA   6449
Heinz1706     ATTATTTTTCTTAGTTTTTTTCATATATTTTTTTAAAAAAAATTCTTTCTTTCTTTTCAGTTTAAAGTTCTTCAAA   6046

Indigo-Rose   ATGAGAATAAAGAATTCATCTCCATATATGAGTATTAGCATAGGTATGTCATTACATGTCAATATTGTTGATAAT   6524
Heinz1706     ATGAGAATAAAGAATTCATCTCCATATATGAGTATTAGCATAGGTATGTCATTACATGTCAATATTGTTGATAAT   6121

Indigo-Rose   CCACCTTCGTTTTCAATTGAGTTATTTCAGGATTTTGGGGTCAATGTAGGGTATATGACAAAACCTAAAAAAGTT   6599
Heinz1706     CCACCTTCGTTTTCAATTGAGTTATTTCAGGATTTTGGGGTCAATGTAGGGTATATGACAAAACCTAAAAAAGTT   6196

Indigo-Rose   CAAGTTGAACAATATCTTGAATAGAGATTCAAGAAAAAAATGACCCAATTACAATTCAGAAAGTCATCAACAACG   6674
Heinz1706     CAAGTTGAACAATATCTTGAATAGAGATTCAAGAAAAAAATGACCCAATTACAATTCAGAAAGTCATCAACAACG   6271

Indigo-Rose   CGAAATCTAGCGGCATTATAGTTGAAGGTTGAAGCAAGAGAAAAGTCAAACATAAATTCAGACGAGATCGGATCT   6749
Heinz1706     CGAAATCTAGCGGCATTATAGTTGAAGGTTGAAGCAAGAGAAAAGTCAAACATAAATTCAGACGAGATCGGATCT   6346

Indigo-Rose   ATCTCATCAAAACTGGACAGAACTAGACAAATCTGAAGAACATCAGGTTGTGTAATTTATATACAAATTACTAAA   6824
Heinz1706     ATCTCATCAAAACTGGACAGAACTAGACAAATCTGAAGAACATCAGGTTGTGTAATTTATATACAAATTACTAAA   6421

Indigo-Rose   GTTATGTATATAGTGGGTTGTGTATTAAGGTAATATATACAAAGTATTATGAGATAATTGTTATTTGTATACAAA   6899
Heinz1706     GTTATGTATATAGTGGGTTGTGTATTAAGGTAATATATACAAAGTATTATGAGATAATTGTTATTTGTATACAAA   6496

Indigo-Rose   TATTTGTATAAAAGAAATTTGTTAATCATTGTATAATACATATATAAAATTATAGTACATTATACAACTTTGTCG   6974
Heinz1706     TATTTGTATAAAAGAAATTTGTTAATCATTGTATAATACATATATAAAATTATAGTACATTATACAACTTTGTCG   6571

Indigo-Rose   ATATATAAAAAAAATTATGAACATGATAAAATATTGTATATATAAACATTACACACTAAATACACAAACATATTT   7049
Heinz1706     ATATATAAAAAAAATTATGAACATGATAAAATATTGTATATATAAACATTACACACTAAATACACAAACATATTT   6646

Indigo-Rose   CAAGTTTACCATATATACAATTGACAAATGAATATTATACAATCATTTTGATCATTAATTTTTTTTAAAAAACAT   7124
Heinz1706     CAAGTTTACCATATATACAATTGACAAACGAATATTATACAATCATTTTGATCATTAATTTTTTTTTAAAAAACAT   6721

Indigo-Rose   GAAATTACATATGTACATATACAAATATGATTCATATACCAAATTATTGTATATTATACAAATCCATACATATAT   7199
Heinz1706     GAAATTACATATGTACATATACAAATATGATTCATATACCAAATTATTGTATATTATACAAATCCATACATATAT   6796

Indigo-Rose   ACTATAAAAAAATGAACATAATAAAATATTGTATACAAATAAACTACAGAAAATAATTAAGAACAAATACAACAC   7274
Heinz1706     ACTATAAAAAAATGAACATAATAAAATATTGTATACAAATAAACTACAGAAAATAATTAAGAACAAATACAACAC   6871

Indigo-Rose   ACTTTTATTTTAAAAAACAAACATAATTGTCATATATACAATTTTCATATTTATGATGAATAATACATAATAAAT   7349
Heinz1706     ACTTTTATTTTAAAAAACAAACATAATTGTCATATATACAATTTTCATATTTATGATGAATAATACATAATAAAT   6946

Indigo-Rose   TACACATGAAATTGTATAAAAAATTATTTACAATTTTTCAAATATATACAATATAATCAATGTGTGTGTGTATAT   7424
Heinz1706     TACACATGAAATTGTATAAAAAATTATTTACAATTTCTCAAATATATACAATATAATCAATGTGTGTGTGTATAT   7021

Indigo-Rose   ATATATATATAAATAAAATCATTAACGTCTAAGTGTTTCATACAACTTATTTTTAAATAAAATTGAGAAAATATT   7499
Heinz1706     ATATATATATAAATAAAATCATTAACGTCTAAGTGTTTCATACAACTTATTTTTAAATAAAATTGAGAAAATATT   7096

Indigo-Rose   TGATTTTGAATGGTTAGCTTAAATAATGGGATTGTGTAAATGTTTGTTACCTTATTTAGCGATATTGTTACCATT   7574
Heinz1706     TGATTTTGAATGGTTAGCTTAAATAATGGGATTGTGTAAATGTTTGTTACCTTATTTAGCGATATTGTTACCATT   7171

Indigo-Rose   CAATGAATAAGTAAAATATTAAATAAATTAAAAATAAAAAAATATTTTTTTACAAATTAAAAGATACATAACAAA   7649
Heinz1706     CAATGAATAAGTAAAATATTAAATAAATTAAAAATAAAAAAATATTTTTTTACAAATTAAAAGATACATAACAAA   7246
```

```
Indigo-Rose   CTAAACTATACCTATAAAATGTAATTATGTAAACTACACCTATATAATATTATTTAATAAATTATGTTTGCTATT   7724
Heinz1706     CTAAACTATACCCATAAAATGTAATTATGTAAACCACACCTATATAATATTATTTAATAAATTATGTTTGCCATT   7321

Indigo-Rose   TATGAAATTTTCCTTATTAATTAATTACACGATTTACAAGCAGTGAGGAACAAAAATTGAGGTGGCAAAACTTAT   7799
Heinz1706     TATGAAATTTTCCTTATTAATTAATTACACGATTTACAAGCAGTGAGGAACAAAAATTGAGGTGGCAAAACTTAT   7396

Indigo-Rose   CCTGATGATGCGGCATGTATAGCTTCATTTTGATGTCACACTAATACTATAGTCTATATATAGTAGCAATACTTG   7874
Heinz1706     CCTGATGATGCGGCATGTATAGCTTCATTTTGATGTCACACTAATACTATAGTCTATATATAGTAGCAATACTTG   7471

Indigo-Rose   ATAGAATCTTCAAATAATCAACCCAACTTAATAAAGTACTAAACAATATTTCGTGTGGATAACTATGAATAGAAT   7949
Heinz1706     ATAGAATCTTCAAATAATCAACCCAACTTAATAACGTACTAAACAATATTTCGTGTGGATAACTATGAATAGAAT   7546

Indigo-Rose   CGGAT   7954
Heinz1706     CGGAT   7551
```

Fig. S2 Sequence polymorphism of the genomic sequence of *SlMYBATV*. Sequences were aligned by using Clustal X ver. 2 (Larkin *et al.*, 2007) with default settings, and conserved nucleotides were shaded by GeneDoc (2.6) (Nicholas *et al.*, 1997).

R2

```
AtMYBL2       --------MN--------------KTR----------LRALSPPSGMQHR-------------KRCRLRGRNY   28
MdMYB6        -MAVSRKDMD--------------RIKGPWSPEEDDSLQRLVQKHGPRNWSLISKSI-PGRSGKSCRLRWCNQ   57
PtrMYB182     -MRKPCCDKR-------------GNNKGAWSTEEDQKLIDYIQTHGEGCWRSIPEAAGLHRCGKSCRLRWINY   59
GhMYB6        -MRKPCCDKQ-------------GTNKGAWSKQEDQKLIDYIRIHGEGCWRSLPKAAGLHRCGKSCRLRWINY   59
PpMYB19       -MRKPCCEKE-------------GTNKGAWSKQEDQKLIDYIKAHGEGCWRSLPKAAGLHRCGKSCRLRWINY   59
VvMYBC2-L3    -MRKPCCDKK-------------DTTKGAWSKQEDQRLIDYIKTHGEGCWRSLPKAAGLHRCGKSCRLRWINY   59
VvMYB4-like   -MRKPCCDKK-------------DTTKGAWSKQEDQRLIDYIKTHGEGCWRSLPKAAGLHRCGKSCRLRWINY   59
VvMYBC2-L1    -MRKPCCDKQ-------------DTNKGAWSKQEDQKLIDYIRKNGEGCWRTLPQAAGLLRCGKSCRLRWINY   59
PpMYB18       -MRKPCCDKQ-------------DTNRGAWSKQEDLKLIDYIRKHGEGCWRTLPQAAGLLRCGKSCRLRWINY   59
GmMYB100      -MRKPSCDIK-------------DLNKGAWSKQEDQKLIDYIKKHGEVCWRTLPQAAGLHRCGKSCRLRWINY   59
TrMYB133      -MRKPSCDIKLE----------KNINKGVWSKQEDQKLIDYINKHGEVCWSTLPQAAGLLRCGKSCRLRWMNY   62
PhMYB27       -MRKACCDNKEE------------MHRGAWSKQEDQKLIDYITKHGAGCWRNLPKADGLLRCGKSCRLRWMNY   60
SlMYB76       -MRKPCCDNKEE------------MHKGAWSKQEDQKLIDYITKHGEGCWRNLPKAAGLLRCGKSCRLRWMNY   60
VvMYBC2-L2    -MRKPAGYGEKKSTKKRVGCEKKFTNKGAWSKQEDQKLIDYIQKHGEGCWSSLPQSAGLLRCGKSCRLRWVNY   72
MtMYB2        -MRKPCCDKE-------------NINKGAWSKQEDQKLIDYIQVHGEGCWGSIPKAAGLHRCGKSCRLRWLNY   59
TrMYB134      -MRTPCCDKE-------------NINKGAWSKQEDKKLIDYIQVHGEGCWGSIPKAAGLHRCGKSCRLRWLNY   59
FaMYB1        -MRKPCCEKT-------------ETTKGAWSIQEDQKLIDYIQKHGEGCWNSLPKAAGLRRCGKSCRLRWINY   59
ZmMYB31       MGRSPCCEKA-------------HTNKGAWTKEEDERLVAHIRAHGEGCWRSLPKAAGLLRCGKSCRLRWINY   60
ZmMYB42       MGRSPCCEKA-------------HTNRGAWTKEEDERLVAYVRAHGEGCWRSLPRAAGLLRCGKSCRLRWINY   60
VvMYB4a       MGRSPCCEKA-------------HTNKGAWTKEEDDRLIAYIRAHGEGCWRSLPKAAGLLRCGKSCRLRWINY   60
PdMYB221      MGRSPCCEKA-------------HTNKGAWTKEEDDRLIAYIRTHGEGCWRSLPKAAGLLRCGKSCRLRWINY   60
PpMYB17       MGRSPCCEKA-------------HTNKGAWTKEEDDRLIAYIRAHGEGCWRSLPKAAGLLRCGKSCRLRWINY   60
AmMYB308      MGRSPCCEKA-------------HTNKGAWTKEEDDRLVAYIRAHGEGCWRSLPKAAGLLRCGKSCRLRWINY   60
CmMYB1        MGRSPCCEKA-------------HTNKGAWTKEEDDRLIAYIRTHGEGCWRSLPKAAGLLRCGKSCRLRWINY   60
PhMYB4        MGRSPCCEKA-------------HTNKGAWTKEEDERLIAYIKAHGEGCWRSLPKAAGLLRCGKSCRLRWINY   60
SlMYB32       MGRSPCCEKA-------------HTNKGAWTKEEDERLISYIRAHGEGCWRSLPKAAGLLRCGKSCRLRWINY   60
SlMYB7        MGRSPCCEKA-------------HTNKGAWTKEEDERLISYIKTHGEGCWRSLPKAAGLLRCGKSCRLRWINY   60
EgMYB1        MGRSPCCEKA-------------HTNKGAWTKEEDDKLIAYIRAHGEGCWRSLPKAAGLLRCGKSCRLRWINY   60
AtMYB32       MGRSPCCEKD-------------HTNKGAWTKEEDDKLISYIKAHGEGCWRSLPRSAGLQRCGKSCRLRWINY   60
AtMYB7        MGRSPCCEKE-------------HMNKGAWTKEEDERLVSYIKSHGEGCWRSLPRAAGLLRCGKSCRLRWINY   60
AtMYB4        MGRSPCCEKA-------------HTNKGAWTKEEDERLVAYIKAHGEGCWRSLPKAAGLLRCGKSCRLRWINY   60
LlMYB1        MGRSPCCEKA-------------HTNKGAWTKEEDDRLVAYIRAHGEGCWRSLPKAAGLLRCGKSCRLRWINY   60
AtMYB3        MGRSPCCEKA-------------HMNKGAWTKEEDQLLVDYIRKHGEGCWRSLPRAAGLQRCGKSCRLRWMNY   60
PpMYB20       MGRSPCCEKA-------------HTNKGAWTKEEDQRLIDYIRVHGEGCWRSLPKAAGLLRCGKSCRLRWINY   60
SlMYB3        MGRSPCCEKA-------------HTNKGAWTKEEDQRLINYIRAHGEGCWRSLPKAAGLSRCGKSCRLRWINY   60
GbMYBF2       MGRQPCCDKAG------------LKKGPWTAEEDRKLVNFITTHGEGCWREVPKLAGLLRCGKSCRLRWTNY   60
MdMYB10       -MEGYNENLS--------------VRKGAWTREEDNLLRQCVEIHGEGKWNQVSYKAGLNRCRKSCRQRWLNY   58
AtETC2        -----------------------------------MDNTNRLRLRRGPSLRQTKFTRSRYDSEEVSSIEWE--   36
AtTRY         -----------------------------------MDNTDRRRRRK-----QHKIA--LHDSEEVSSIEWE--   29
VvTRY         -----------------------------------MD----RRRRK-----LPKIA-APSQSEEVSSIEWE--   26
SlTRY         -----------------------------------MDQNLHHRHKL----MHHRCC---SHEEEVNSMEWE--   29
AtETC3        -----------------------------------MDNHRRTKQPK----TN-SIVTSSSEGTEVSSLEWE--   31
AtETC1        -----------------------------------MNTQRKSKHLK----TNPTIVASSSE--EVSSLEWE--   30
AtCPC         -------------------------------MFRSDKAEKMDKRRR----RQSKAKASCSE--EVSSIEWE--   34
EcROI1        -----------------------------------MAS-SENSSSDD-YFSTSQE-------VNKKETKV---   26
ElROI1        -----------------------------------MAS-SENSSSDD-YFSTSQE-------VNKKESKV---   26
PhMYBx        -----------------------------------MADKGQSSSSVN-TPADSQDGVAPRMLVSGKTSKVA--   35
SlMYBATV-X1   -----------------------------------MADWNRSSTSDN-ASVVSPDSTRVVA-LETTNEETS--   34
SlMYBATV-X3   -----------------------------------MADWNRSSTSDN-ASVVSPDSTRVVA-LETTNEETS--   34
SlMYBATV-X2   -----------------------------------MADWNRSSTSDN-ASVVSPDSTRVVA-LETTNEETS--   34
GmMYB73       -----------------------------------MADIDR-SFDNN-VSAVSTEKS----------SQVS--   24
SlMYBATV-like -----------------------------------MADSDSSSTSNY-AFIDSPPFEAK-------KEESL--   28
```

R3/bHLH-binding C1

```
AtMYBL2        VRPEVKQRNFSKDEDDLIIKLHALLGNRWSLIAGRLPGRTDNEVRIHWETYLKRKLVKMG-IDPTN----HRL   96
MdMYB6         LSPQVEHRAFTPEEDDMIIRAHARFGNKWATIARLLNGRTDNAIKNHWNSTLKRKCSDGGGVDLNGGYDGHFL  130
PtrMYB182      LRPDIKRGNFGQDEEDLIIKLHALLGNRWSLIAGRLPGRTDNEVKNYWNSHLKKKLIDMG-IDPNNHR-LNQI  130
GhMYB6         LRPDIKRGNFAQDEEDLIIKLHALLGNRWSLIAGRLPGRTDNEVKNYWNSHIKRKLMKMG-IDPNNHK-LNQY  130
PpMYB19        LRPDIKRGNFEQDEEDLIIKLHALLGNRWSLIAGRLPGRTDNEVKNYWNSHIRKKLIKMG-IDPNNHR-LNQI  130
VvMYBC2-L3     LRPDLKRGNFGQDEEDLIIKLHALLGNRWSLIAGRLPGRTDNEVKNYWNSHIRKKLINMG-IDPNNHR-VNQS  130
VvMYB4-like    LRPDLKRGNFGQDEEDLIIKLHALLGNRWSLIAGRLPGRTDNEVKNYWNSHIRKKLINMG-IDPNNHR-VNQS  130
VvMYBC2-L1     LRPDLKRGDFAEDEEDLIIKLHALLGNRWSLIAGRPPGRTDNEVKNYWNSHLRKKLINMG-IDPNNHR-LSHN  130
PpMYB18        LRPDLKRGNFAEDEEDLIVKLHALLGNRWSLIAGRLPGRTDNEVKNYWNSHLRKKLISMG-IDPNNHRPNTFN  131
GmMYB100       LRPDLKRGNFAEDEEDLIIKLHALLGNRWSLIAGRLPGRTDNEVKNYWNSHIRRKLISKG-IDPNNHR-LKHT  130
TrMYB133       LRPDVKRGNFGEDEEDLIIKLHALLGNRWSLIAGRLPGRTDNEVKNFWNSRIRKKLIRKG-IDPNNHN-LHHK  133
PhMYB27        LSPNLKRGNFSEDEEDLIIKLHALLGNRWSLIAGRLPGRTDNEVKNYWNSHLRRKLIKMG-IDPKNHR-----  127
SlMYB76        LNPNLKRGNFSEDEDDLIIKLHALLGNRWSLIAGRLPGRTDNEVKNYWNSHLTRKLIKMG-IDPKNHR-----  127
VvMYBC2-L2     LKPDVKRGNFGEDEEDLIIKLHALLGNRWSLIAGRLPGRTDNEVKNYWNSHLKKKLMRMG-IDPNNHR-----  139
MtMYB2         LRPDIKRGIFAQDEEDLIIKLHALLGNRWALIAGRLPGRTDNEVKNYWNSHIRRKLIKMG-IDPNNHK-LHKG  130
TrMYB134       LRPDIKRGIFAQDEEDLIIKLHALLGNRWALIAGRLPGRTDNEVKNYWNSHIRRKLIKMG-IDPNNHK-LHKG  130
FaMYB1         LRPDLKRGSFGEDEEDLIIRLHKLLGNRWSLIAGRLPGRTDNEVKNYWNSHLKKKILKTG-TTLRPNK-----  126
ZmMYB31        LRPDLKRGNFTEEEDELIVKLHSVLGNKWSLIAGRLPGRTDNEIKNYWNTHIRRKLLSRG-IDPVTHRPVTEH  132
ZmMYB42        LRPDLKRGNFTADEDDLIVKLHSLLGNKWSLIAARLPGRTDNEIKNYWNTHIRRKLLGSG-IDPVTHRRVAGG  132
VvMYB4a        LRPDLKRGNFTEEEDELIIKLHSLLGNKWSLIAGRLPGRTDNEIKNYWNTHIRRKLLNRG-IDPSTHRPINE-  131
PdMYB221       LRPDLKRGNFTEEEDELIIKLHSLLGNKWSLIAGRLPGRTDNEIKNYWNTHIRRKLLNRG-IDPATHRPLNE-  131
PpMYB17        LRPDLKRGNFTEEEDELIIKLHSLLGNKWSLIAGRLPGRTDNEIKNYWNTHIRRKLLTRG-IDPTTHRALNE-  131
AmMYB308       LRPDLKRGNFTEEEDELIIKLHSLLGNKWSLIAGRLPGRTDNEIKNYWNTHIRRKLLSRG-IDPTTHRSINDG  132
CmMYB1         LRPDLKRGNFTEDEDELIIKLHSLLGNKWSLIAGRLPGRTDNEIKNYWNTHIRRKLLNRG-IDPATHRPVTDH  132
PhMYB4         LRPDLKRGNFTEDEDELIIKLHSLLGNKWSLIAGRLPGRTDNEIKNYWNTHIRRKLLSRG-IDPTTHRIMNE-  131
SlMYB32        LRPDLKRGNFTEEEDELIIKLHSLLGNKWSLIAGRLPGRTDNEIKNYWNTHIRRKLLSRG-IDPTTHRSIND-  131
SlMYB7         LRPDLKRGNFTEEEDELIIKLHSLLGNKWSLIAGRLPGRTDNEIKNYWNTHIRRKLLSRG-IDPTTHRPVNE-  131
EgMYB1         LRPDLKRGNFTEEEDEIIIKLHSLLGNKWSLIAGRLPGRTDNEIKNYWNTHIRRKLLNRG-IDPATHRLINEP  132
AtMYB32        LRPDLKRGNFTLEEDDLIIKLHSLLGNKWSLIATRLPGRTDNEIKNYWNTHVKRKLLRKG-IDPATHRPINET  132
AtMYB7         LRPDLKRGNFTHDEDELIIKLHSLLGNKWSLIAARLPGRTDNEIKNYWNTHIKRKLLSKG-IDPATHRGINEA  132
AtMYB4         LRPDLKRGNFTEEEDELIIKLHSLLGNKWSLIAGRLPGRTDNEIKNYWNTHIRRKLINRG-IDPTSHRPIQES  132
LlMYB1         LRPDLKRGNFTEEEDELIIKLHSLLGNKWSLIAGRLPGRTDNEMKNYWNTHIRRKLLSRG-IDPTTHRPLNDA  132
AtMYB3         LRPDLKRGNFTEEEDELIIKLHSLLGNKWSLIAGRLPGRTDNEIKNYWNTHIKRKLLSRG-IDPNSHRLIN--  130
PpMYB20        LRPDLKRGNFTEEEDELIIKLHSLLGNKWSLIAGRLPGRTDNEIKNYWNTHIKRKLISRG-LDPQTHRPLN--  130
SlMYB3         LRPDLKRGNFTEEEDELIIKLHSLLGNKWSVIAGRLPGRTDNEIKNYWNTHIKRKLISRG-IDPQTHRPLNNN  132
GbMYBF2        LRPDLKRGLLSESEEKLIIELHAAIGNRWSRIAAQLPGRTDNEIKNYWNTRIKKKLRQMG-IDPVTHKPLNQM  132
MdMYB10        LKPNIKRGDFKEDEVDLIIRLHRLLGNRWSLIARRLPGRTANAVKNYWNTRLRIDSRMKTVKNKSQEMRKTNV  131
AtETC2         ------FISMTEQEEDLISRMYRLVGNRWDLIAGRVVGRKANEIERYWIMRNSDYFSHKRRRLNNSPFFSTSP  103
AtTRY          ------FINMTEQEEDLIFRMYRLVGDRWDLIAGRVPGRQPEEIERYWIMRNSEGFADKRRQLHSSSHKHTKP   96
VvTRY          ------FINMSEQEEDLIYRMYRLVGDRWDLIAGRIPGRKPEEIERFWIMRNRQGYAERGKEASQKKQ-----   88
SlTRY          ------FIRMSKQEEDLIYRMHKLVGDRWGLIAGRIPGRTAEEIERFWIMRHSDGFAHKRRQTIKKSLPPT--   94
AtETC3         ------VVNMSQEEEDLVSRMHKLVGDRWELIAGRIPGRTAGEIERFWVMKN---------------------   77
AtETC1         ------EIAMAQEEEDLICRMYKLVGERWDLIAGRIPGRTAEEIERFWVMKN------HRRSQLR--------   83
AtCPC          ------AVKMSEEEEDLISRMYKLVGDRWELIAGRIPGRTPEEIERYWLMKHGVVFANRRRDFFRK-------   94
EcROI1         ------QVQLSKDEEDLVIRMYNLVGDRWPLIAGRIPGRSADQIKKYWLSRYSTTAFPKQKINR---------   84
ElROI1         ------QVQLSEDEEDLVIRMYNLVGDRWPLIAGRIPGRSADQIKKYWLSRYSTTAFPKQKINR---------   84
PhMYBx         ------EIKFSEEEEDLIIRMYNLVGERWSLIAGRIPGRSAEEIEKYWNTRSSTSQ-----------------   85
SlMYBATV-X1    ------KLEFSEDEEMLIAKMFSLVRERWSLIAGRIPGRNADEIEKYWKSKYSKSQEEIQAQSQDEAHGIRLI  101
SlMYBATV-X3    ------KLEFSEDEEMLIAKMFSLVRERWSLIAGRIPGRNADEIEKYWKSKYSKSQ-----------------   84
SlMYBATV-X2    ------KLEFSEDEEMLIAKMFSLVRERWSLIAGRIPGRNADEIEKYWKSKYSKSQ--ILGSM----------   89
GmMYB73        ------DVEFSEAEEILIAMVYNLVGERWSLIAGRIPGRTAEEIEKYWTSRFSTSQ-----------------   74
SlMYBATV-like  ------KLEFSQDEEILITKMFNLVGERWSLIAGRIQGELQMKLRSTGTQEIPTANKIFSFCYIWSYKRKK--   93
```

```
AtMYBL2         HHHTNYISRRHLHS--------------------SHKEHETKIISDQSSSVS---------------------   128
MdMYB6          RDHEQPPLKRSVSAGSGVPVSTGLYMSPGSPSGSDASDSSAQVMSLSDCHVYRPL------------------   185
PtrMYB182       LPRLQAEP-AAPVIATST----------------TTGSKNNVAASKPKNLSD---------------------   165
GhMYB6          P--HHVGP-LSPITTNSM----------------DVACKLRVFSTE---------------------------   157
PpMYB19         IPRPNPQNDCVSAAATSS----------------GSMSNISACTKAPIKSSR---------------------   166
VvMYBC2-L3      F----AAPLNRCASAATM----------------SSGSMTGACDNKSLKPS----------------------   161
VvMYB4-like     F----AAPLNRCASAATM----------------SSGSMTGACDNKSLKPS----------------------   161
VvMYBC2-L1      FPRPRDPCTAATAT--------------------SSGLNNHAS-PPVKS-VG---------------------   160
PpMYB18         LPRPHHKNSQAISSTAKL----------------SAGLKTPTNDQPARS-GG---------------------   166
GmMYB100        IPSS--LQNSLMSDDSSK----------------AFSMKDTNKNETSKLPRV---------------------   164
TrMYB133        IPP---LQNQIMSN-SLK----------------YFGLKEISKNETTKT-HL---------------------   164
PhMYB27         -----------ISHYLH------------------RKRLEYWSENSS---RG---------------------   147
SlMYB76         -----------LSHYLH------------------IKRLELLQENNT---RL---------------------   147
VvMYBC2-L2      -----------LGERASG----------------TSKSFESRDQTSNPLISA---------------------   164
MtMYB2          FPT---------VGTSS------------------CVESMNKENNKLS-IKS---------------------   154
TrMYB134        FPSP---HHVFAAGTSSS----------------SCDKERNINNLTLIKSKS---------------------   163
FaMYB1          -----------------------------------PHENNHAPNNKLVKLFN---------------------   143
ZmMYB31         HASNITISFETEVAAAARDD--------------KKGAVFRLEEEEERNKAT---------------------   170
ZmMYB42         AATTISFQPSPNTAVAAAAE--------------TAAQAPIKAEETAAVKAP---------------------   170
VvMYB4a         PSPDV--TTISFA-----------------------AAVKEE-EKINISSTG---------------------   157
PdMYB221        PAQEAS-TTISFSTTTSVKEE-------------SLSSVKEESNKEKIISAA---------------------   169
PpMYB17         AAQDSATTTISFAAS---------------------ANIKEEDQKSSIIN-----------------------   160
AmMYB308        TASQDQVTTISFSNANSKE---------------EDTKHKVAVDIMIKEE-----------------------   167
CmMYB1          PTTTTTTTTTVATTVNNNHNVTSPPPDIISFATTKTTDHVVKNEDMEQETK----------------------   183
PhMYB4          PSTQ-KVTTISFAAGNEDIK---------------DQKISIKAEFEQIK------------------------   164
SlMYB32         PTTIPKVTTITFAAAHENIKDIDQ----------QDEMINIKAEFVETSKES---------------------   173
SlMYB7          PGTTQKVTTISFAGGDHKTKDIEED---------HNKMINVKAESGLSQ------------------------   171
EgMYB1          AQDHHDEPTISFAANSKE-----------------IKEMKNNAELNFMCN-----------------------   165
AtMYB32         KTSQDSSDSSKTEDPLVK----ILSFG---PQLEKIANFG--DERIQKRV-----------------------   173
AtMYB7          KIS----DLKKTKDQIVK----DVSFV---TKFEETDKSG--DQKQNKYIRN---------------------   171
AtMYB4          SASQ---DSKPTQLEPVTSNTINISFTS-APKVETFHESISFPGKSEKISML---------------------   180
LlMYB1          SQNQSHASSASAAKQHDS------------------EILITDTDANSKRP-----------------------   164
AtMYB3          --ESVVSPSSLQNDVVETIH-------------LDFSGPVKPEPVREEIG---------MVNNCES-------   172
PpMYB20         --DTTTAAAAAA-ATTPASR-------------LDFRNISPPSAVVDKTTNKNSILHHKTINKTSSNNNNHNS   187
SlMYB3          ATNSHTTTNITTAVTTKNIN-------------LDFTNVDQKQPNIMIATSS-------SYDETKCN------   179
GbMYBF2         QMDYMIRPHAPMNAEENNKFEP------------PLEPENVAEASSPENIRG---------------------   172
MdMYB10         IRPQPQKFNRSSYYLSSKEP-----------ILDHIQSAEDLSTPPQTSSSTK--------------------   173
AtETC2          LNLQENLKL----------------------------------------------------------------   112
AtTRY           HRPRFSIYPS---------------------------------------------------------------   106
VvTRY           -------------------------------------------------------------------------     -
SlTRY           -------------------------------------------------------------------------     -
AtETC3          -------------------------------------------------------------------------     -
AtETC1          -------------------------------------------------------------------------     -
AtCPC           -------------------------------------------------------------------------     -
EcROI1          -------------------------------------------------------------------------     -
ElROI1          -------------------------------------------------------------------------     -
PhMYBx          -------------------------------------------------------------------------     -
SlMYBATV-X1     EKTGPSTGHPQQCVFPEKKPISVCQPSDIGPQLLGPTTNGPQQLLGPKTTGPNNITVDQKLVNPAQDQINQ--   172
SlMYBATV-X3     -------------------------------------------------------------------------     -
SlMYBATV-X2     -------------------------------------------------------------------------     -
GmMYB73         -------------------------------------------------------------------------     -
SlMYBATV-like   -------------------------------------------------------------------------     -
```

C2/EAR

```
AtMYBL2        -------------ESCGVTILPIPSTNCSEDSTSTG------RSHLPDLNIGL-IPAVTS-------------   168
MdMYB6         -------------ARTGGVLPPAETTSSSNNNSSNS------EKNDPPTSLSLSLPGVDSEEVS---------   230
PtrMYB182      ----------------GDSDRVSDTATCLEDDYESLVTQQAATSGSSSINIDLNIAAPASPGHR---------   213
GhMYB6         -----------------NDDGVSDAASYLEDETP--------PTGVSNLDLDLTIAFPSSP-IK---------   195
PpMYB19        ----------------EIDQRASQATSVLEDETS--------GSSSRDLNLDLTIAFPNPPLLV---------   206
VvMYBC2-L3     ----------------ADNDPLSDSASGLEDEIS----------ASLDLNLDLTIAIPASS-LT---------   198
VvMYB4-like    ----------------ADNDPLSDSASGLEDEIS----------ASLDLNLDLTIAIPASS-LT---------   198
VvMYBC2-L1     ----------------DN-DQTSDAGSCLDDN----------RRALPDLNLDVAITIPQPS--V---------   195
PpMYB18        ----------------NNCDQVSDGTSCLEDESC--------GHQLPDLNLDLTMTAPLSNSEP---------   206
GmMYB100       ----------------YNHVEVSDAASGEAES----------PCALPDLNLDLSITNPSAS-QS---------   201
TrMYB133       ----------------DNYDKVSNATSGNKDE----------SYALLDLNLELSL------------------   193
PhMYB27        ----------------TDHEVVSDAGSSCAKHQ---------PSSLPDLNS-----PPSIH------------   178
SlMYB76        ----------------ENVGVISDATSSYANKDQQI------TSSLLDLNL-----IP---------------   178
VvMYBC2-L2     ----------------ADNNAVLDSTCGSASKT---------TSSLPDLNLNLNVGAPSVD------------   200
MtMYB2         ----------------CDAP-INNDVSFTKKDTT------SIINSSSSLNLDLTIALPSPNR-----------   193
TrMYB134       ----------------CEEEYINSSVSFTKKETS------IINNSSSSLNLELTIALPSPN------------   202
FaMYB1         ----------------KMDDEVVDEVSSADSAAG---------CLVPELNLDLTLSIKTST------------   179
ZmMYB31        ----------------MVVGRDRQSQSQSHSHPAGEWGQGKRPLKCPDLNLDLCISPPCQEE----EEMEEAA   223
ZmMYB42        --------------------------------------------RCPDLNLDLCISPPCQHEDDGEEEEEELD   199
VvMYB4a        ----------------GFGCKTEKNPVTEKC---------------PDLNLELRISPPY--Q-------PQAE   190
PdMYB221       ----------------AFICKEEKTPVQERC---------------PDLNLELRISLPCQNQ-------PDRH   204
PpMYB17        ----------------GLLGKDSKKPVQERC---------------PDLNLELQISPPCQ-P-------QQPS   194
AmMYB308       ---------------------NSP--VQERC---------------PDLNLDLKISPPCQQQ------INYHQ   196
CmMYB1         ------------------LNITSPDQKQERC---------------PDLNLELRIGPPQHHSSNMIPSISYMQ   223
PhMYB4         -----------------DDEIISK-PIKEQC---------------PDLNLELKISPPYQQH-------SDRA   197
SlMYB32        ----------------DNNEIIQE-KSSSCL---------------PDLNLELRISPPHHQQ-------LDHH   207
SlMYB7         ----------------LEDEIISSSPFREQC---------------PDLNLELRISPPSLQN-------YQHS   206
EgMYB1         ----------------LEESADVASSARERC---------------PDLNLELGISPPSHQL-------HQPE   200
AtMYB32        ----------------------EYSVVEERC---------------LDLNLELRISPPWQDKL----------   199
AtMYB7         ----------------GLVCKEERVVVEEKIG--------------PDLNLELRISPPWQNQ-----------   203
AtMYB4         ----------------TFKEEKDECPVQEKF---------------PDLNLELRISLPDDVDR----------   212
LlMYB1         -------------------------GVFEVC---------------PDLNLELTISLPHHHQ-----------   186
AtMYB3         ------------------SGTTSEKDYGNEED--------------WVLNLELSVGPS-YRYES-----TRKV   207
PpMYB20        NIVLFKPKMEEDNIEEASSGTTTEEDQQQQQQQQQQKLQDHYMYKCSDLNLDLSIGLEPFQSEP-----TRAS   255
SlMYB3         ------------------SGTTEETKPLEIIIPKIP--------SQVMINLELSIGLPLHTDHI-----SSPE   221
GbMYBF2        ----------------PDEEMGMNMPACSSLESLEIDLPLRMEDSMPLLNLCLPISVISLPGRTEEVDSDDSN   229
MdMYB10        ----------------NGNDWWETLLEGEDTFER---------AAYPSIELEEELFTSFWFDDR---------   212
AtETC2         -------------------------------------------------------------------------     -
AtTRY          -------------------------------------------------------------------------     -
VvTRY          -------------------------------------------------------------------------     -
SlTRY          -------------------------------------------------------------------------     -
AtETC3         -------------------------------------------------------------------------     -
AtETC1         -------------------------------------------------------------------------     -
AtCPC          -------------------------------------------------------------------------     -
EcROI1         -------------------------------------------------------------------------     -
ElROI1         -------------------------------------------------------------------------     -
PhMYBx         -------------------------------------------------------------------------     -
SlMYBATV-X1    -------------------------------------------------------------------------     -
SlMYBATV-X3    -------------------------------------------------------------------------     -
SlMYBATV-X2    -------------------------------------------------------------------------     -
GmMYB73        -------------------------------------------------------------------------     -
SlMYBATV-like  -------------------------------------------------------------------------     -
```

```
AtMYBL2         ------------------LPALCLQDSSESSTNGSTGQETLLLFR----------------------------  195
MdMYB6          ------------------NQVAPVTESTQAPAPAPVSTTSVAVQNIPVELLAVMQGMIRKEVRNYMAGLEQSG  285
PtrMYB182       -------------------TTFGNNQQNCKWGKTSQVERDPSSL-----------------------------  238
GhMYB6          -------------------NIIEESQQKTASIVTNDEE-EQYTV-----------------------------  219
PpMYB19         -------------------GEEMQKNIKGSFTMAREIETNLQHS-----------------------------  231
VvMYBC2-L3      -------------------DVIDKKRQDTKSIFSREVEGDAN-------------------------------  221
VvMYB4-like     -------------------DVIDKKRQDTKSIFSREVEGDAN-------------------------------  221
VvMYBC2-L1      -------------------DTTEEAKKHNEPKVSRELEPGPS-------------------------------  218
PpMYB18         -------------------ENLKEEQNLMSLKCHRNLPRPPI-------------------------------  229
GmMYB100        -------------------ISGNNVKPFHESNSSRKVQFDSP-------------------------------  224
TrMYB133        -------------------------------------------------------------------------    -
PhMYB27         -----------------------SSCAQP--------------------------------------------  184
SlMYB76         -------------------------------------------------------------------------    -
VvMYBC2-L2      -----------------------EQMQLTGANSHKELEPAPF-------------------------------  219
MtMYB2          -----------------------IVIPNCESPKTRDMDIDLNC------------------------------  213
TrMYB134        ---------------------------DCESPKIRDIDIDLNC------------------------------  218
FaMYB1          ------------------------------GMADPQVA-----------------------------------  187
ZmMYB31         MRVRPAVKREA---------GLCFGCSLGLPR-TADCKCSS------------------------SSFLGLR-  261
ZmMYB42         LIKPAVVKREALQAGHGHGHGLCLGCGLGGQKGAAGCSCSNG-----------------------HHFLGLR-  248
VvMYB4a         TPLKTGGRSSS--------TTLCFACSLGIPN-SEECSCS------------IGTSSGS--SSSGYDFLGL--  238
PdMYB221        QAFKTGG---S--------TSLCFACSLGLQN-SKDCSCSVIVG-------TIGSSSSAG-SKTGYDFLGM--  255
PpMYB17         EPLKSGG------------RGVCFSCSLGLQD-AKNCSCG--ID-------TIGSSTTSGTTNVGYDFLGL--  243
AmMYB308        ENLKTGGRNGS--------STLCFVCRLGIQN-SKDCSCSDG--------------VGN--------------  232
CmMYB1          QPLMTGG-------------TICFACSLGVQD-NKECRCSTNGT------------SGNNNSNVGFDFFKI--  268
PhMYB4          LQQSTTGSGGA--------STICFTCSLGLKN-NKGCSCSRNR---------------SMN-VAGYDFLGLK-  244
SlMYB32         RHHQRS-------------SSLCFTCSLGIQN-SKDCSCGSESNGN-----GWSNNMVSMNIMAGYDFLGLK-  260
SlMYB7          -------------------PSRCFACSLGIQN-SKDCNCSKNN-------------------IASYNFLGLK-  239
EgMYB1          PLLRFTGR--K--------SDLCXECNLGLKN-SQNCRCSVG--------------VIESETSVGYDFLGL--  246
AtMYB32         HDERNLRFGRV--------KYRCSACRFGFGN-GKECSCNNVKCQTEDSSSSSYSSTDISSS-IGYDFLGLNN  262
AtMYB7          ---------RE--------ISTCTASRFYMEN-DMECSSETVKCQTENSSSISYSSIDISSSNVGYDFLGLK-  257
AtMYB4          LQGHG---KST--------TPRCFKCSLGMIN-GMECRCGRMRCDVVGGSS---KGSDMSNG---FDFLGLAK  267
LlMYB1          -------------------HQQPDQISLKSIN-SESSTAGDG-----------------------FDFLESK-  215
AtMYB3          SVVDSAESTRR-------WGSELFG----AHESDAVCLCCRIG------------------------LFRNES  245
PpMYB20         SGNSAESRLQQ-------NNYQVFGTVHKAGVTQAVCLCCQVG------------------------FQSSDA  297
SlMYB3          STASYNFLTTV-------APPPTAAVPAAEMMAKTVCLCWQIG------------------------YHGGGG  263
GbMYBF2         IVDQVN-------------NPLSDSHKNNANNNMTTSCCSFNNGGN---IHSRSSDIKAETPSLWGDQMGVLG  286
MdMYB10         ---------------------LSPRSCANFPEGQSRSEFSFS-------------------------------  233
AtETC2          -------------------------------------------------------------------------    -
AtTRY           -------------------------------------------------------------------------    -
VvTRY           -------------------------------------------------------------------------    -
SlTRY           -------------------------------------------------------------------------    -
AtETC3          -------------------------------------------------------------------------    -
AtETC1          -------------------------------------------------------------------------    -
AtCPC           -------------------------------------------------------------------------    -
EcROI1          -------------------------------------------------------------------------    -
ElROI1          -------------------------------------------------------------------------    -
PhMYBx          -------------------------------------------------------------------------    -
SlMYBATV-X1     -------------------------------------------------------------------------    -
SlMYBATV-X3     -------------------------------------------------------------------------    -
SlMYBATV-X2     -------------------------------------------------------------------------    -
GmMYB73         -------------------------------------------------------------------------    -
SlMYBATV-like   -------------------------------------------------------------------------    -
```

TLLLFR

```
AtMYBL2        ------------------------------------------------------------   -
MdMYB6         VCFQQAAAGNEGFWNVGVKRIGFSRIE---------------------------------   312
PtrMYB182      ---PTLLLFR--------------------------------------------------   245
GhMYB6         ---PTLLLFR--------------------------------------------------   226
PpMYB19        ---PTLALFR--------------------------------------------------   238
VvMYBC2-L3     ---PTLILFR--------------------------------------------------   228
VvMYB4-like    ---PTLILFR--------------------------------------------------   228
VvMYBC2-L1     ---STLLLFG--------------------------------------------------   225
PpMYB18        ---SFLS-----------------------------------------------------   233
GmMYB100       ---STLLLFQ--------------------------------------------------   231
TrMYB133       ------------------------------------------------------------   -
PhMYB27        ------------------------------------------------------------   -
SlMYB76        ------------------------------------------------------------   -
VvMYBC2-L2     ---TTLLLFG--------------------------------------------------   226
MtMYB2         ------------------------------------------------------------   -
TrMYB134       ------------------------------------------------------------   -
FaMYB1         ------------------------------------------------------------   -
ZmMYB31        ---TAMLDFRSLEMK---------------------------------------------   273
ZmMYB42        ---TSVLDFRGLEMK---------------------------------------------   260
VvMYB4a        --TSGVLDYRGLEMK---------------------------------------------   251
PdMYB221       --KSGVLDYRGLEMK---------------------------------------------   268
PpMYB17        --KSGVLDYRSLEMK---------------------------------------------   256
AmMYB308       ------------------------------------------------------------   -
CmMYB1         --KNGALDYRSLEMK---------------------------------------------   281
PhMYB4         --TNG-LDYRTLETRTK-------------------------------------------   258
SlMYB32        --TNGLLDYRTLETK---------------------------------------------   273
SlMYB7         --SNGVLDYRTLETK---------------------------------------------   252
EgMYB1         --KASVLDYRS-------------------------------------------------   255
AtMYB32        ---TRVLDFSTLEMK---------------------------------------------   274
AtMYB7         ---TRILDFRSLEMK---------------------------------------------   269
AtMYB4         KETTSLLGFRSLEMK---------------------------------------------   282
LlMYB1         ---SSVLDCRSSVTQIDWRALTE-------------------------------------   235
AtMYB3         --CRNCRVSD--VRTH--------------------------------------------   257
PpMYB20        --CRNCQCTNGFYRFHRPLNS---------------------------------------   316
SlMYB3         QWCGKCKNTNGFYRYC--------------------------------------------   279
GbMYBF2        DPISDYLQWNDLELWSAGLDAASGWIQQLPECNWSEMQGDIEISSNCPELQRLASILDEM   346
MdMYB10        ---TDLWNHSKEE-----------------------------------------------   243
AtETC2         ------------------------------------------------------------   -
AtTRY          ------------------------------------------------------------   -
VvTRY          ------------------------------------------------------------   -
SlTRY          ------------------------------------------------------------   -
AtETC3         ------------------------------------------------------------   -
AtETC1         ------------------------------------------------------------   -
AtCPC          ------------------------------------------------------------   -
EcROI1         ------------------------------------------------------------   -
ElROI1         ------------------------------------------------------------   -
PhMYBx         ------------------------------------------------------------   -
SlMYBATV-X1    ------------------------------------------------------------   -
SlMYBATV-X3    ------------------------------------------------------------   -
SlMYBATV-X2    ------------------------------------------------------------   -
GmMYB73        ------------------------------------------------------------   -
SlMYBATV-like  ------------------------------------------------------------   -
```

Fig. S3 Multiple sequence alignment of MYB flavonoid repressor based on its full-length proteins. Entire protein sequences were aligned by using Clustal X ver. 2 (Larkin *et al.*, 2007) with default settings, and the conserved amino acids were shaded by using GeneDoc (2.6) (Nicholas *et al.*, 1997). The R2, R3/bHLH-binding, C1, C2/EAR, and TLLLFR domains (or motifs) are marked above the alignment.

R2

```
MtMYB2         -MRKPC-------------CDKE---NINKGAWSKQEDQKLIDYIQVHGEGCWGSIPKAAGLHRCGKSCRLRWL   57
TrMYB134       -MRTPC-------------CDKE---NINKGAWSKQEDKKLIDYIQVHGEGCWGSIPKAAGLHRCGKSCRLRWL   57
PtrMYB182      -MRKPC-------------CDKR---GNNKGAWSTEEDQKLIDYIQTHGEGCWRSIPEAAGLHRCGKSCRLRWI   57
GhMYB6         -MRKPC-------------CDKQ---GTNKGAWSKQEDQKLIDYIRIHGEGCWRSLPKAAGLHRCGKSCRLRWI   57
PpMYB19        -MRKPC-------------CEKE---GTNKGAWSKQEDQKLIDYIKAHGEGCWRSLPKAAGLHRCGKSCRLRWI   57
VvMYBC2-L3     -MRKPC-------------CDKK---DTTKGAWSKQEDQRLIDYIKTHGEGCWRSLPKAAGLHRCGKSCRLRWI   57
VvMYB4-like    -MRKPC-------------CDKK---DTTKGAWSKQEDQRLIDYIKTHGEGCWRSLPKAAGLHRCGKSCRLRWI   57
VvMYBC2-L1     -MRKPC-------------CDKQ---DTNKGAWSKQEDQKLIDYIRKNGEGCWRTLPQAAGLLRCGKSCRLRWI   57
PpMYB18        -MRKPC-------------CDKQ---DTNRGAWSKQEDLKLIDYIRKHGEGCWRTLPQAAGLLRCGKSCRLRWI   57
GmMYB100       -MRKPS-------------CDIK---DLNKGAWSKQEDQKLIDYIKKHGEVCWRTLPQAAGLHRCGKSCRLRWI   57
TrMYB133       -MRKPS-------------CDIKLEKNINKGVWSKQEDQKLIDYINKHGEVCWSTLPQAAGLLRCGKSCRLRWM   60
PhMYB27        -MRKAC-------------CDNK--EEMHRGAWSKQEDQKLIDYITKHGAGCWRNLPKADGLLRCGKSCRLRWM   58
SlMYB76        -MRKPC-------------CDNK--EEMHKGAWSKQEDQKLIDYITKHGEGCWRNLPKAAGLLRCGKSCRLRWM   58
FaMYB1         -MRKPC-------------CEKT---ETTKGAWSIQEDQKLIDYIQKHGEGCWNSLPKAAGLRRCGKSCRLRWI   57
VvMYBC2-L2     -MRKPAGYGEKKSTKKRVGCEKK---FTNKGAWSKQEDQKLIDYIQKHGEGCWSSLPQSAGLLRCGKSCRLRWV   70
VvMYB4a        MGRSPC-------------CEKA---HTNKGAWTKEEDDRLIAYIRAHGEGCWRSLPKAAGLLRCGKSCRLRWI   58
PpMYB17        MGRSPC-------------CEKA---HTNKGAWTKEEDDRLIAYIRAHGEGCWRSLPKAAGLLRCGKSCRLRWI   58
EgMYB1         MGRSPC-------------CEKA---HTNKGAWTKEEDDKLIAYIRAHGEGCWRSLPKAAGLLRCGKSCRLRWI   58
CmMYB1         MGRSPC-------------CEKA---HTNKGAWTKEEDDRLIAYIRTHGEGCWRSLPKAAGLLRCGKSCRLRWI   58
PdMYB221       MGRSPC-------------CEKA---HTNKGAWTKEEDDRLIAYIRTHGEGCWRSLPKAAGLLRCGKSCRLRWI   58
AmMYB308       MGRSPC-------------CEKA---HTNKGAWTKEEDDRLVAYIRAHGEGCWRSLPKAAGLLRCGKSCRLRWI   58
LlMYB1         MGRSPC-------------CEKA---HTNKGAWTKEEDDRLVAYIRAHGEGCWRSLPKAAGLLRCGKSCRLRWI   58
PhMYB4         MGRSPC-------------CEKA---HTNKGAWTKEEDERLIAYIKAHGEGCWRSLPKAAGLLRCGKSCRLRWI   58
AtMYB4         MGRSPC-------------CEKA---HTNKGAWTKEEDERLVAYIKAHGEGCWRSLPKAAGLLRCGKSCRLRWI   58
ZmMYB31        MGRSPC-------------CEKA---HTNKGAWTKEEDERLVAHIRAHGEGCWRSLPKAAGLLRCGKSCRLRWI   58
ZmMYB42        MGRSPC-------------CEKA---HTNRGAWTKEEDERLVAYVRAHGEGCWRSLPRAAGLLRCGKSCRLRWI   58
SlMYB7         MGRSPC-------------CEKA---HTNKGAWTKEEDERLISYIKTHGEGCWRSLPKAAGLLRCGKSCRLRWI   58
SlMYB32        MGRSPC-------------CEKA---HTNKGAWTKEEDERLISYIRAHGEGCWRSLPKAAGLLRCGKSCRLRWI   58
PpMYB20        MGRSPC-------------CEKA---HTNKGAWTKEEDQRLIDYIRVHGEGCWRSLPKAAGLLRCGKSCRLRWI   58
SlMYB3         MGRSPC-------------CEKA---HTNKGAWTKEEDQRLINYIRAHGEGCWRSLPKAAGLSRCGKSCRLRWI   58
AtMYB3         MGRSPC-------------CEKA---HMNKGAWTKEEDQLLVDYIRKHGEGCWRSLPRAAGLQRCGKSCRLRWM   58
AtMYB7         MGRSPC-------------CEKE---HMNKGAWTKEEDERLVSYIKSHGEGCWRSLPRAAGLLRCGKSCRLRWI   58
PtrMYB57       MGRSPC-------------CEKE---HTNKGAWTKEEDERLINYIKSHGEGCWRSLPKAAGLLRCGKSCRLRWI   58
AtMYB32        MGRSPC-------------CEKD---HTNKGAWTKEEDDKLISYIKAHGEGCWRSLPRSAGLQRCGKSCRLRWI   58
GbMYBF2        MGRQPC-------------CDKA---GLKKGPWTAEEDRKLVNFITTHGEGCWREVPKLAGLLRCGKSCRLRWT   58
MdMYB10        MEGYN---------------ENL---SVRKGAWTREEDNLLRQCVEIHGEGKWNQVSYKAGLNRCRKSCRQRWL   56
MdMYB6         MAVSRK--------------DMD----RIKGPWSPEEDDSLQRLVQKHGPRNWSLISKSIP-GRSGKSCRLRWC   55
AtMYBL2        -----------------------------------------------MNKTRLRALSPPSG-MQHRKRCRLRGR   26
AtETC2         ----------------------------------------MDNTNRLRLRRGPSLRQTKFTRSRYDS------E   28
AtTRY          ----------------------------------------MDNTDRRRRR-----KQHKIA--LHDS------E   21
VvTRY          ----------------------------------------MD----RRRR-----KLPKIA-APSQS------E   18
SlTRY          ----------------------------------------MDQNLHHRHK----LMHHRCC---SHE------E   21
AtETC3         ----------------------------------------MDNHRRT-KQP---KTN-SIVTSSSEG------T   23
AtETC1         ----------------------------------------MNTQRKS-KHL---KTNPTIVASSSE--------   22
AtCPC          -------------------------------------MFRSDKAEKMDKRR---RRQSKAKASCSE--------   26
EcROI1         ----------------------------------------MAS-SENSSSD-------DYFSTSQE-------V   19
ElROI1         ----------------------------------------MAS-SENSSSD-------DYFSTSQE-------V   19
PhMYBx         ----------------------------------------MADKGQSSSSV-------NTPADSQDGVAPRMLV   27
SlMYBATV-X2    ----------------------------------------MAD-WNRSSTS-------DNASVVSPDSTRVVAL   26
SlMYBATV-X3    ----------------------------------------MAD-WNRSSTS-------DNASVVSPDSTRVVAL   26
SlMYBATV-X1    ----------------------------------------MAD-WNRSSTS-------DNASVVSPDSTRVVAL   26
SlMYBATV-like  ----------------------------------------MAD-SDSSSTS-------NYAFIDSP------PF   20
GmMYB73        ----------------------------------------MAD-IDRSFDN-------NVSAVSTE--------   18
```

R3/bHLH-binding

```
MtMYB2          NYLRPDIKRGIFAQDEEDLIIKLHALLGNRWALIAGRLPGRTDNEVKNYWNSHIR   112
TrMYB134        NYLRPDIKRGIFAQDEEDLIIKLHALLGNRWALIAGRLPGRTDNEVKNYWNSHIR   112
PtrMYB182       NYLRPDIKRGNFGQDEEDLIIKLHALLGNRWSLIAGRLPGRTDNEVKNYWNSHLK   112
GhMYB6          NYLRPDIKRGNFAQDEEDLIIKLHALLGNRWSLIAGRLPGRTDNEVKNYWNSHIK   112
PpMYB19         NYLRPDIKRGNFEQDEEDLIIKLHALLGNRWSLIAGRLPGRTDNEVKNYWNSHIR   112
VvMYBC2-L3      NYLRPDLKRGNFGQDEEDLIIKLHALLGNRWSLIAGRLPGRTDNEVKNYWNSHIR   112
VvMYB4-like     NYLRPDLKRGNFGQDEEDLIIKLHALLGNRWSLIAGRLPGRTDNEVKNYWNSHIR   112
VvMYBC2-L1      NYLRPDLKRGDFAEDEEDLIIKLHALLGNRWSLIAGRPPGRTDNEVKNYWNSHLR   112
PpMYB18         NYLRPDLKRGNFAEDEEDLIVKLHALLGNRWSLIAGRLPGRTDNEVKNYWNSHLR   112
GmMYB100        NYLRPDLKRGNFAEDEEDLIIKLHALLGNRWSLIAGRLPGRTDNEVKNYWNSHIR   112
TrMYB133        NYLRPDVKRGNFGEDEEDLIIKLHALLGNRWSLIAGRLPGRTDNEVKNFWNSRIR   115
PhMYB27         NYLSPNLKRGNFSEDEEDLIIKLHALLGNRWSLIAGRLPGRTDNEVKNYWNSHLR   113
SlMYB76         NYLNPNLKRGNFSEDEDDLIIKLHALLGNRWSLIAGRLPGRTDNEVKNYWNSHLT   113
FaMYB1          NYLRPDLKRGSFGEDEEDLIIRLHKLLGNRWSLIAGRLPGRTDNEVKNYWNSHLK   112
VvMYBC2-L2      NYLKPDVKRGNFGEDEEDLIIKLHALLGNRWSLIAGRLPGRTDNEVKNYWNSHLK   125
VvMYB4a         NYLRPDLKRGNFTEEEDELIIKLHSLLGNKWSLIAGRLPGRTDNEIKNYWNTHIR   113
PpMYB17         NYLRPDLKRGNFTEEEDELIIKLHSLLGNKWSLIAGRLPGRTDNEIKNYWNTHIR   113
EgMYB1          NYLRPDLKRGNFTEEEDEIIIKLHSLLGNKWSLIAGRLPGRTDNEIKNYWNTHIR   113
CmMYB1          NYLRPDLKRGNFTEDEDELIIKLHSLLGNKWSLIAGRLPGRTDNEIKNYWNTHIR   113
PdMYB221        NYLRPDLKRGNFTEEEDELIIKLHSLLGNKWSLIAGRLPGRTDNEIKNYWNTHIR   113
AmMYB308        NYLRPDLKRGNFTEEEDELIIKLHSLLGNKWSLIAGRLPGRTDNEIKNYWNTHIR   113
LlMYB1          NYLRPDLKRGNFTEEEDELIIKLHSLLGNKWSLIAGRLPGRTDNEMKNYWNTHIR   113
PhMYB4          NYLRPDLKRGNFTEDEDELIIKLHSLLGNKWSLIAGRLPGRTDNEIKNYWNTHIR   113
AtMYB4          NYLRPDLKRGNFTEEEDELIIKLHSLLGNKWSLIAGRLPGRTDNEIKNYWNTHIR   113
ZmMYB31         NYLRPDLKRGNFTEEEDELIVKLHSVLGNKWSLIAGRLPGRTDNEIKNYWNTHIR   113
ZmMYB42         NYLRPDLKRGNFTADEDDLIVKLHSLLGNKWSLIAARLPGRTDNEIKNYWNTHIR   113
SlMYB7          NYLRPDLKRGNFTEEEDELIIKLHSLLGNKWSLIAGRLPGRTDNEIKNYWNTHIR   113
SlMYB32         NYLRPDLKRGNFTEEEDELIIKLHSLLGNKWSLIAGRLPGRTDNEIKNYWNTHIR   113
PpMYB20         NYLRPDLKRGNFTEEEDELIIKLHSLLGNKWSLIAGRLPGRTDNEIKNYWNTHIK   113
SlMYB3          NYLRPDLKRGNFTEEEDELIIKLHSLLGNKWSVIAGRLPGRTDNEIKNYWNTHIK   113
AtMYB3          NYLRPDLKRGNFTEEEDELIIKLHSLLGNKWSLIAGRLPGRTDNEIKNYWNTHIK   113
AtMYB7          NYLRPDLKRGNFTHDEDELIIKLHSLLGNKWSLIAARLPGRTDNEIKNYWNTHIK   113
PtrMYB57        NYLRPDLKRGNFSDEEDELIINLHSLLGNKWSLIAARLPGRTDNEIKNYWNTHIK   113
AtMYB32         NYLRPDLKRGNFTLEEDDLIIKLHSLLGNKWSLIATRLPGRTDNEIKNYWNTHVK   113
GbMYBF2         NYLRPDLKRGLLSESEEKLIIELHAAIGNRWSRIAAQLPGRTDNEIKNYWNTRIK   113
MdMYB10         NYLKPNIKRGDFKEDEVDLIIRLHRLLGNRWSLIARRLPGRTANAVKNYWNTRLR   111
MdMYB6          NQLSPQVEHRAFTPEEDDMIIRAHARFGNKWATIARLLNGRTDNAIKNHWNSTLK   110
AtMYBL2         NYVRPEVKQRNFSKDEDDLILKLHALLGNRWSLIAGRLPGRTDNEVRIHWETYLK    81
AtETC2          EVSSIEWEFISMTEQEEDLISRMYRLVGNRWDLIAGRVVGRKANEIERYWIMRNS    83
AtTRY           EVSSIEWEFINMTEQEEDLIFRMYRLVGDRWDLIAGRVPGRQPEEIERYWIMRNS    76
VvTRY           EVSSIEWEFINMSEQEEDLIYRMYRLVGDRWDLIAGRIPGRKPEEIERFWIMRNR    73
SlTRY           EVNSMEWEFIRMSKQEEDLIYRMHKLVGDRWGLIAGRIPGRTAEEIERFWIMRHS    76
AtETC3          EVSSLEWEVVNMSQEEEDLVSRMHKLVGDRWELIAGRIPGRTAGEIERFWVMKN-    77
AtETC1          EVSSLEWEEIAMAQEEEDLICRMYKLVGERWDLIAGRIPGRTAEEIERFWVMKNH    77
AtCPC           EVSSIEWEAVKMSEEEEDLISRMYKLVGDRWELIAGRIPGRTPEEIERYWLMKHG    81
EcROI1          NKKETKV-QVQLSKDEEDLVIRMYNLVGDRWPLIAGRIPGRSADQIKKYWLSRYS    73
ElROI1          NKKESKV-QVQLSEDEEDLVIRMYNLVGDRWPLIAGRIPGRSADQIKKYWLSRYS    73
PhMYBx          SGKTSKVAEIKFSEEEEDLIIRMYNLVGERWSLIAGRIPGRSAEEIEKYWNTRSS    82
SlMYBATV-X2     ETTNEETSKLEFSEDEEMLIAKMFSLVRERWSLIAGRIPGRNADEIEKYWKSKYS    81
SlMYBATV-X3     ETTNEETSKLEFSEDEEMLIAKMFSLVRERWSLIAGRIPGRNADEIEKYWKSKYS    81
SlMYBATV-X1     ETTNEETSKLEFSEDEEMLIAKMFSLVRERWSLIAGRIPGRNADEIEKYWKSKYS    81
SlMYBATV-like   EAKKEESLKLEFSQDEEILITKMFNLVGERWSLIAGRIQGELQMKLRSTGTQEIP    75
GmMYB73         --KSSQVSDVEFSEAEEILIAMVYNLVGERWSLIAGRIPGRTAEEIEKYWTSRFS    71
```

Fig. S4 Multiple sequence alignment of MYB flavonoid repressor based on the R3 and R2R3 domains. The N-terminal protein sequences comparing the R2R3 domain or only the R3 domain for small MYB proteins were aligned by using Clustal X ver. 2 (Larkin *et al.*, 2007) with default settings, and the conserved amino acids were shaded by using GeneDoc (2.6) (Nicholas *et al.*, 1997). The R2 and R3/bHLH-binding domains are marked above the alignment.

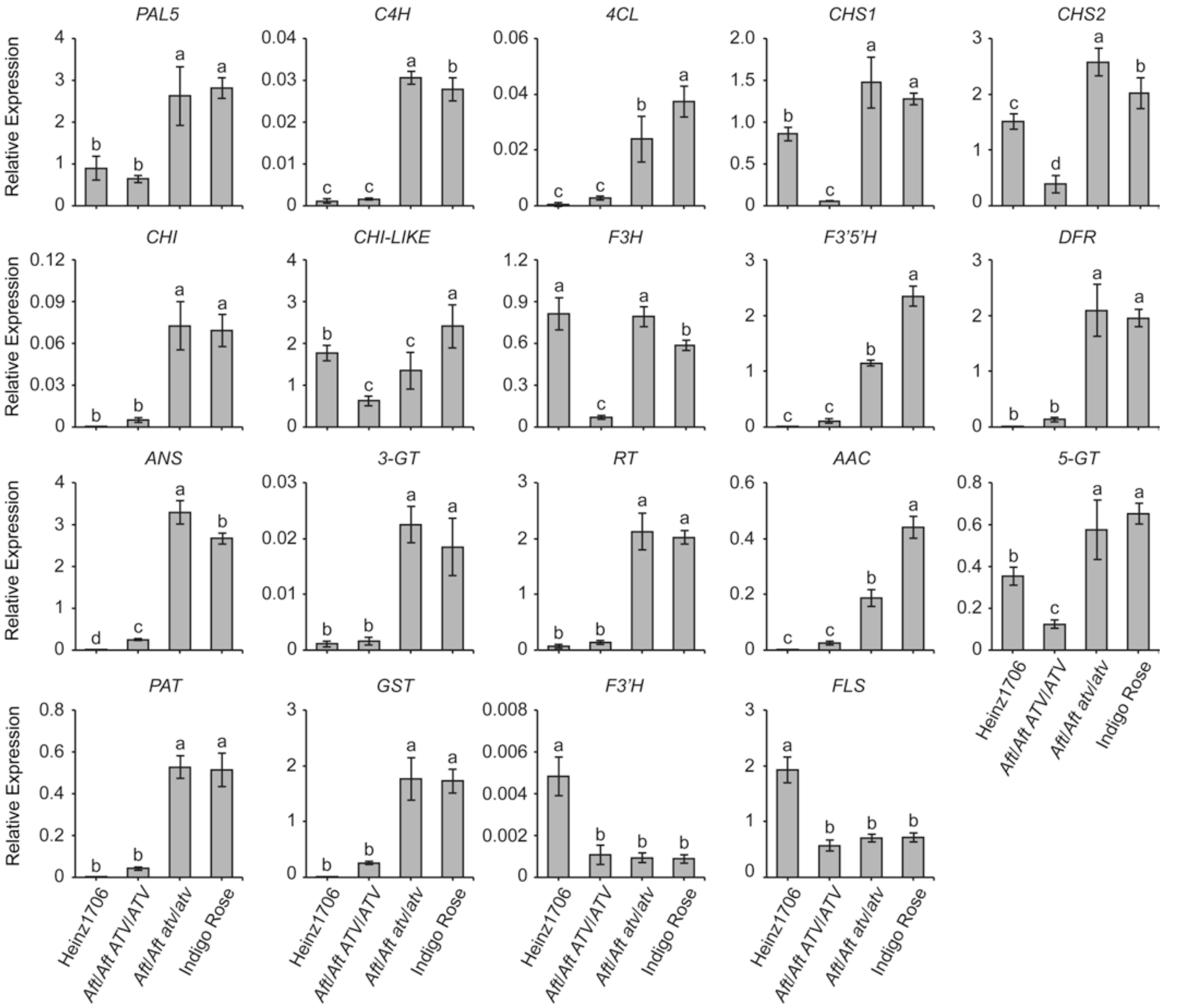

Fig. S5 Transcriptional analysis of the structural genes of the anthocyanin biosynthetic pathway in the peel of tomato fruit at the fully ripened stage. Transcriptional profiling of these genes was determined by qRT-PCR in the fruit peel of *Aft*/*Aft ATV*/*ATV* (abbreviated as *Aft*/*Aft*) and *Aft*/*Aft atv*/*atv* in a F5 population, and the two parent lines Heinz1706 (genotype, *AFT*/*AFT ATV*/*ATV*) and Indigo Rose (*Aft*/*Aft atv*/*atv*). A tomato *ACTIN* (*Solyc03g078400*) gene was used as the reference: relative expression levels are shown. Different letters indicate statistically significant differences among groups (Tukey's honestly significant difference test, $P<0.01$). PAL, phenyl alanine ammonia-lyase; C4H, cinnamate-4-hydroxylase; 4CL, 4-coumaroyl:CoA-ligase; CHS, chalcone synthase; CHI, chalcone isomerase; CHI-LIKE, chalcone isomerase-like; F3H, flavanone 3-hydroxylase; F3´5´H, flavonoid 3´5´-hydroxylase; DFR, dihydroflavonol 4-reductase; ANS, leucoanthocyanidin dioxygenase; 3-GT, 3-O-glucosyltransferase; RT, rhamnosyl transferase; AAC, anthocyanin acyltransferase; 5-GT, 5-O-glucosyltransferase; PAT, putative anthocyanin transporter; GST, glutathione S-transferase; F3´H, flavonoid 3´-hydroxilase; FLS, flavonol synthase.

Table S1. General information on the DNA markers used in this study

Marker name	Genome position	Forward primer	Reverse primer	Marker type	Restriction enzyme	Heinz1706 allele (bp)	Indigo Rose allele (bp)
ATV-In	SL2.50ch07: 61,003,572	GAGGTTTCTTCGTTGGTAGTC	CTAAATAAAAGTTATTGAGTTCACG	InDel		85	89
HP1865	SL2.50ch07: 25,351,236	TTATGCCAAAGCTTCAACGG	ACGAGTTTATTTTAAGTTTCCTTG	InDel		158	176
HP1877	SL2.50ch07: 59,804,770	CACGGGAATAAGAAATTGGCG	TGGTTGACTCTTGAAGTTTCAGT	InDel		148	171
HP1885	SL2.50ch07: 62,676,901	AGGTACTTGTGACCAAACCC	TTCAGCATGAACTGACTGGG	InDel		136	157
HP1891	SL2.50ch07: 64,799,803	AGCTTTGCTAAGACATACTAAGAC	ACTGTTTTCGATAGATCCTCGG	InDel		151	137
HP1915	SL2.50ch07: 60,726,148	CTTAAGAAGGAAGTATGAAGAGAG	CCATCAATCATAGTTTCACCG	InDel		97	108
HP1917	SL2.50ch07: 60,982,128	TCACAAGTAAAGGAGCATCC	CTCTTCTTATAGTCACGTAAGTTAG	InDel		138	117
HP1919	SL2.50ch07: 61,321,298	CTCTCAACGATTCTATCTTCAC	CCCAATTTGATATCTGAACC	InDel		119	132
HP1949	SL2.50ch10: 65,012,425	TGTGGCACCTCCACCGTAAC	CCGCATGACCTATAAGCGAC	InDel		124	154
HP1953	SL2.50ch10: 65,134,020	TTCTTAGTAACCGCGATGCTC	GACTTTGCTCCAGATGAAGTG	InDel		768	376
HP2675	SL2.50ch07: 60,920,438	CTGTTGATGGTAAACACTTTTCTC	CAAAAGTTTCATAAAAGCAGACC	InDel		148	160
HP2677	SL2.50ch07: 61,044,268	GTGTTACCAAAAAAAGGAAAGTG	GATCATTGCTTCTAGAAATAGGC	InDel		131	143
HP3195	SL2.50ch10: 65,177,657	GAGTAAACATATAAGCATTCCTTTG	CAATCACTAAGTAGTTGTTGCTCA	InDel		89	69
HP3217	SL2.50ch07: 61,013,666	ACATTGGTGGCATTGAATATGAG	ACAAGTATCACCCTATCCGTCTC	InDel		200	236
JP13	SL2.50ch07: 60,995,265	CTGGTATAATCTCCCTTTAGCG	AAGGTCTAAGAAAAGCTGACAAC	CAPS	MseI	176	128
JP17	SL2.50ch07: 61,004,897	GTTGTATAAGGCGGAGTGATGGCGAC	GCATCTGCGCATATTCTCTTC	dCAPS	HinfI	148	172

Table S2. Primers used for sequencing and cDNA cloning

Primer Name	Forward primer (5'->3')	Reverse primer (5'->3')	Notes
ATVg-1	CCGAAGTTAACAAATACACGAAC	CGAGATCGGATCTATCTCATCA	Sequencing *SlMYBATV* gene
ATVg-2	ATATCGACAAAGTTGTATAATGTACTAT	CAACTTTCCAATTATACACACTCG	Sequencing *SlMYBATV* gene
ATVg-3	AGCAATTGTTCGAGGAGAGG	CTTTCAGAAGTTCGTGTATCTTGC	Sequencing *SlMYBATV* gene
ATVg-4	GTAAATGTGAAGATCCTGAAATGAC	ATGATATGATTATCTGCGTCTAACC	Sequencing *SlMYBATV* gene
ATVg-5	GAACATGTATTTTGGAAGTAGCC	CTTCTCGCACCTCTTCACTG	Sequencing *SlMYBATV* gene
ATVg-6	GCATGTCGAGTTAATGAGGATG	AAGGAAGTAGTGGAAATCCTACAAG	Sequencing *SlMYBATV* gene
ATVg-7	GGGTTTGGGCTCATTGGTGT	AGGATCAGAAATTAGTTGCCAC	Sequencing *SlMYBATV* gene
3RACE-RT	AAGCAGTGGTATCAACGCAGAGTACTTTTTTTTTTTTTTTTTTTTTTTTTTTTTVN		3'-RACE CDS Primer, Clontech
SlAN2	GAATACTCCTATGTGTGCATCG	CCAACTAAGGATATGTCATAGTAATAAG	Clonging *SlAN2* cDNA
SlAN2-like	TGCAGTATTTGTGACTTAACATATC	GGTATTGTAGTACTACTTGTACTAGCA	Clonging *SlAN2-like* cDNA
SlMYB76	CTTCTTCACATAGTCACCATCC	AAGCAGTGGTATCAACGCAGAGT (3RACE-NUP, Clontech)	Clonging *SlMYBATV*-like cDNA
SlMYBATV	GACCACATTCAAATCTTTGTTC	AAGCAGTGGTATCAACGCAGAGT (3RACE-NUP, Clontech)	Clonging *SlMYBATV*-like cDNA
SlMYBATV-like	TGCCAAGTTATTTCCATCTC	AAGCAGTGGTATCAACGCAGAGT (3RACE-NUP, Clontech)	Clonging *SlMYBATV*-like cDNA
SlTRY	CAGAAGCTGTCCCTCCTAATC	GCAACTCTCTGCTAGTCCTCT	Clonging *SlTRY* cDNA

Table S3. Information regarding the known MYB repressors for the phenylpropanoid and flavonoid pathway

Name	Full Name	Type	GenBank Accession Number	Reference
AmMYB308	*Antirrhinum majus* MYB308	R2R3 MYB	P81393	Jackson *et al.*, 1991
AtCPC	*Arabidopsis thaliana* CAPRICE	R3 MYB	BAA21917	Wada *et al.*, 1997
AtETC1	*Arabidopsis thaliana* Enhancer of TRY and CPC 1	R3 MYB	NP_171645	Kirik *et al.*, 2004a
AtETC2	*Arabidopsis thaliana* Enhancer of TRY and CPC 2	R3 MYB	NP_850145	Kirik *et al.*, 2004b
AtETC3	*Arabidopsis thaliana* Enhancer of TRY and CPC 2	R3 MYB	NP_192015	Simon *et al.*, 2007
AtMYB3	*Arabidopsis thaliana* MYB3	R2R3 MYB	Q9S9K9	Kranz *et al.*, 1998
AtMYB4	*Arabidopsis thaliana* MYB4	R2R3 MYB	Q9SZP1	Kranz *et al.*, 1998
AtMYB7	*Arabidopsis thaliana* MYB7	R2R3 MYB	NP_179263	Fornale *et al.*, 2014
AtMYB32	*Arabidopsis thaliana* MYB32	R2R3 MYB	O49608	Preston *et al.*, 2004
AtMYBL2	*Arabidopsis thaliana* MYB-Like 2	R3 MYB	AEE35154	Dubos *et al.*, 2008; Matsui *et al.*, 2008
AtTRY	*Arabidopsis thaliana* TRIPTYCHON	R3 MYB	Q8GV05	Schnittger *et al.*, 1999
CmMYB1	*Chrysanthemum x morifolium* MYB1	R2R3 MYB	AEO27497.1	Zhu *et al.*, 2013
EcROI1	*Erythranthe cardinalis* rose intensity 1	R3 MYB	AGC66792.1	Yuan *et al.*, 2013
EgMYB1	*Eucalyptus gunnii* MYB1	R2R3 MYB	CAE09058.1	Legay *et al.*, 2010
ElROI1	*Erythranthe lewisii* rose intensity 1	R3 MYB	AGC66791.1	Yuan *et al.*, 2013
FaMYB1	*Fragaria x ananassa* MYB1	R2R3 MYB	AAK84064	Aharoni *et al.*, 2001
GbMYBF2	*Ginkgo biloba* MYBF2	R2R3 MYB	AFD36431	Xu *et al.*, 2014
GhMYB6	*Gossypium hirsutum* MYB6	R2R3 MYB	AAN28286	Cedroni *et al.*, 2003
GmMYB73	*Glycine max* MYB73	R3 MYB	ABH02868	Liu *et al.*, 2014
GmMYB100	*Glycine max* MYB100	R2R3 MYB	Glyma07g33960.1	Yan *et al.*, 2015
LlMYB1	*Leucaena leucocephala* MYB1	R2R3 MYB	ADY38393	Omer *et al.*, 2013
MdMYB6	*Malus domestica* MYB6	R2R3 MYB	AAZ20429	Gao *et al.*, 2011
MdMYB10	*Malus domestica* MYB10	R2R3 MYB	ACQ45201	Lin-Wang *et al.*, 2011
MtMYB2	*Medicago truncatula* MYB2	R2R3 MYB	AES99346	Jun *et al.*, 2015
PdMYB221	*Populus trichocarpa* MYB221	R2R3 MYB	ACN97176	Tang *et al.*, 2015
PhMYB4	*Petunia* x *hybrid* MYB4	R2R3 MYB	ADX33331	Colquhoun *et al.*, 2011
PhMYB27	*Petunia* x *hybrid* MYB27	R2R3 MYB	AHX24372	Albert *et al.*, 2011

PhMYBx	*Petunia* x *hybrid* MYBx	R3 MYB	AHX24371	Kroon, 2004
PpMYB17	*Prunus persica* MYB17	R2R3 MYB	ALO81020	Zhou *et al.*, 2016
PpMYB18	*Prunus persica* MYB18	R2R3 MYB	ALO81021	Zhou *et al.*, 2016
PpMYB19	*Prunus persica* MYB19	R2R3 MYB	ALO81022	Zhou *et al.*, 2016
PpMYB20	*Prunus persica* MYB20	R2R3 MYB	ALO81023	Zhou *et al.*, 2016
PtrMYB57	*Populus trichocarpa* MYB57	R2R3 MYB	XP_002325733	Wan *et al.*, 2017
PtrMYB182	*Populus tremula* x *Populus tremuloides* MYB182	R2R3 MYB	AJI76863	Yoshida *et al.*, 2015
Tr-MYB133	*Trifolium repens* MYB133	R2R3 MYB	AMB27081	Albert, 2015
Tr-MYB134	*Trifolium repens* MYB134	R2R3 MYB	AMB27082	Albert, 2015
VvMYBC2-L1	*Vitis vinifera* MYBC2-Like 1	R2R3 MYB	ABW34393	Huang *et al.*, 2014
VvMYBC2-L2	*Vitis vinifera* MYBC2-Like 2	R2R3 MYB	ACX50288	Cavallini *et al.*, 2015
VvMYBC2-L3	*Vitis vinifera* MYBC2-Like 3	R2R3 MYB	AIP98385	Cavallini *et al.*, 2015
VvMYB4a	*Vitis vinifera* MYB4a	R2R3 MYB	ABL61515	Jun *et al.*, 2015
VvMYB4-like	*Vitis vinifera* MYB4-like	R2R3 MYB	XP_002273328.1	Perez-Diaz *et al.*, 2016
VvTRY	*Vitis vinifera* TRIPTYCHON	R3 MYB	ABW34395	Jun *et al.*, 2015
ZmMYB31	*Zea mays* MYB31	R2R3 MYB	NP_001105949	Fornale *et al.*, 2010
ZmMYB42	*Zea mays* MYB42	R2R3 MYB	NP_001106009	Sonbol *et al.*, 2009

Table S4. Primers for quantitative RT-PCR

Gene name	Gene ID	Forward primer	Reverse primer	Reference
3GT	*Solyc10g083440*	CGATCGAACGACGAAACACTG	GCAGCATAGATGGCATTGGC	Developed in this study
4CL	*Solyc06g068650*	TTCACCTCAGGGATATCATGCTCATC	CATGTGAACAAGGTGAAGGATTATGC	Developed in this study
5GT	*Solyc09g092500*	GTGGCATTTCCTCATTGGAC	TCATCACTCTCAACCACACCA	Qiu *et al*., 2016
AAC	*Solyc12g088170*	CTTGCTGGAAGGTTGTCTGAAGATG	CAAGGTCACTATCTTTGTATGCTTTGTC	Developed in this study
ANS	*Solyc08g080040*	GAACTAGCACTTGGCGTCGAA	TTGCAAGCCAGGCACCATA	Qiu *et al*., 2016
C4H	*Solyc05g047530*	AAGAAGGAAACTAATGGCTGAAA	GCTGCAACATTGATATTCTCCA	Developed in this study
CHI	*Solyc05g010320*	CGGCGCAGGGAATAGAGGTTT	CCGTCAAGGGCAAGATCATAGTCAC	Developed in this study
CHI-LIKE	*Solyc05g052240*	TTGATGGGCCATGGAATAACAGATA	GTCAACTGGAGCATTCACAATAGCCT	Developed in this study
CHS1	*Solyc09g091510*	TGGTCACCGTGGAGGAGTATC	GATCGTAGCTGGACCCTCTGC	Povero *et al*., 2011
CHS2	*Solyc05g053550*	GGCGACTCCTTCGAACTGTGTTG	ATCCAAAGAAGGAGCCATGTATTCAC	Developed in this study
DFR	*Solyc02g085020*	AGTCCAAGGATCCAGAGAACGAAGTA	TGGACATCAAGAGTTCCAGCAGAT	Developed in this study
F3'5'H	*Solyc11g066580*	GGTACATGTGGGATGGTTGTTGC	ACTTCCAACGTGGTCCATAGGG	Developed in this study
F3'H	*Solyc03g115220*	ACCACCATTAACTTGAACTCCTCC	GCCATGTCCGACAGGAAGAAG	Developed in this study
F3H	*Solyc02g083860*	GTGAAAAGTTGATGGATTTGGC	GTAATGGTTCCTGGATCGGTGTGT	Qiu *et al*., 2016
FLS	*Solyc11g013110*	CATACAGGGAAGCAAATGAGGAA	AGTATGAGCCACAACTCCAAGAGC	Developed in this study
GST	*Solyc02g081340*	TGCTTTTACAGCCATTTGGACAGG	CCGGTTAGTTTCTTTCCCTTGTCTTC	Developed in this study
PAL	*Solyc03g071870*	GGTGGTGCTCTTCAAAAGGAAC	GGAATGCTTCATGAGCGTCAAGTT	Developed in this study
PAL5	*Solyc09g007900*	GGAATTGCAGGGTTGCCACTTT	AAGGCCGCGTTGCCTAAAGAAG	Povero *et al*., 2011
PAT	*Solyc03g025190*	CGGTGTTTCAGTCCCTCCTA	TTGCATCTCCTTGCTGTTTG	Povero *et al*., 2011
RT	*Solyc09g059170*	CTGGCAATGCAAACAGAGTGA	TCGACTTGCGGAAGAGTGAGA	Qiu *et al*., 2016
SlACTIN	*Solyc03g078400*	GGGATGGAGAAGTTTGGTGGTGG	CTTCGACCAAGGGATGGTGTAGC	Qiu *et al*., 2016
SlAN1	*Solyc09g065100*	CTAAGAGTGCCCGCATACAGAC	ATCCGAAGTGGAGTGCTCAGATA	Qiu *et al*., 2016
SlAN2	*Solyc10g086250*	CCAGCTCTAGCAGGAACAAGATG	AACTCTTAGGCAATAGGTGGTCACT	Developed in this study
SlAN2-like	*Solyc10g086290*	CAAGCTTCTAGGCAATAGATGGTCACT	TTGGTCGTGGTCGTAGTATAGTGC	Developed in this study
SlAN11	*Solyc03g097340*	CCGTCTCTGGGATGTTACTGATACT	CTTCTCGGCTCCACTTCATTCC	Developed in this study
SlANT1	*Solyc10g086260*	AAGCTCTTAGGCAACAGATGGTCAC	TGAGAAATACTTGCGTCGTTGAGG	Developed in this study
SlANT1-like	*Solyc10g086270*	AGGTGACTTCGCTTCGGATGA	CGTTTACTCTTAAACTTTTCACGTGG	Developed in this study
SlJAF13	*Solyc08g081140*	TCAGGGGATCACTACCGAAC	TCCCATCAAGGTTGGAAGAC	Kiferle *et al*.,2015
SlMYB3	*Solyc06g065100*	AGCTGCAGGATTGTCAAGATGTG	GAGCACGTATATAATTGATGAGGCG	Developed in this study
SlMYB7	*Solyc01g111500*	CCTCCTTGGTAACAAATGGTCACT	TTGGATCAATGCCCCGACTC	Developed in this study

SlMYB12	*Solyc01g079620*	ATGCCGGATTATTGAGATGCG	GCTATCAACTTTTCGACTTAGATGAGAG	Developed in this study
SlMYB32	*Solyc10g055410*	ACAAGTGGTCGCTTATAGCAGGAA	TCATGAGCAGCAGCAAAAGTAATC	Developed in this study
SlMYB76	*Solyc05g008250*	CTGCTGGCCTGCTTCGTTGT	CCGTTCGTCCTGGCAATCTTC	Developed in this study
SlMYBATV-like	*Solyc12g005800*	CCTTCATAGACTCCCCTCCATTTG	GACCACCTCTCACCAACCAAGTT	Developed in this study
SlMYBATV-X1	*Solyc07g052490*	TCAGCTTGGTTAGAGAGAGGTG	AAAACACACTGCTGAGGGTGC	Developed in this study
SlMYBATV-X2	*Solyc07g052490*	TCAGCTTGGTTAGAGAGAGGTG	CGCTAGATTTCGCGTTGTTGA	Developed in this study
SlMYBATV-X3	*Solyc07g052490*	TCAGCTTGGTTAGAGAGAGGTG	CATCTGAGGGAAAAAGTAATTTGAAG	Developed in this study
SlTRY	*Solyc01g095640*	GCAGCCATGAAGAAGAGGTTAATAGTAT	TATAAGCCCCCACCTGTCTCCA	Developed in this study

Table S5. Preliminary mapping of the *atv* locus

HP1865[a]	HP1877	HP1885	HP1891	Plant number[b]	Fruit color[c]
3	3	3	3	7	Fully purple
1	2	3	3	1	Fully purple
1	3	3	3	1	Fully purple
2	2	3	3	2	Fully purple
2	3	2	2	4	Fully purple
2	3	3	2	1	Fully purple
2	3	3	3	3	Fully purple
3	3	2	2	2	Fully purple
3	3	3	2	3	Fully purple
1	1	1	1	12	Purple spots
2	2	2	2	25	Purple spots
1	1	1	2	3	Purple spots
1	1	1	3	1	Purple spots
1	1	2	2	6	Purple spots
1	1	2	3	1	Purple spots
1	2	1	1	1	Purple spots
1	2	2	2	2	Purple spots
1	2	2	3	1	Purple spots
1	2	3	3	4	Purple spots
2	1	1	1	7	Purple spots
2	1	2	2	1	Purple spots
2	2	1	1	9	Purple spots
2	2	2	1	3	Purple spots
2	2	2	3	3	Purple spots
2	2	3	2	1	Purple spots
2	2	3	3	3	Purple spots
3	1	1	1	3	Purple spots
3	2	1	1	1	Purple spots
3	2	2	1	1	Purple spots
3	2	2	2	4	Purple spots
3	2	2	3	1	Purple spots
3	3	2	2	4	Purple spots

a Marker score on parent plant: 1, homozygous Heinz1706; 2, heterozygous; 3, homozygous Indigo Rose.

b The number of plants with same phenotype and genotype.

c Purple spots or Fully purple was exhibited on the fruit peel which was exposed to high light.

Reference

Aharoni A, De Vos CH, Wein M, Sun Z, Greco R, Kroon A, Mol JN, O'Connell AP. 2001. The strawberry FaMYB1 transcription factor suppresses anthocyanin and flavonol accumulation in transgenic tobacco. *Plant Journal* **28**, 319-332.

Albert NW. 2015. Subspecialization of R2R3-MYB Repressors for Anthocyanin and Proanthocyanidin Regulation in Forage Legumes. *Front Plant Sci* **6**, 1165.

Albert NW, Lewis DH, Zhang H, Schwinn KE, Jameson PE, Davies KM. 2011. Members of an R2R3-MYB transcription factor family in Petunia are developmentally and environmentally regulated to control complex floral and vegetative pigmentation patterning. *Plant Journal* **65**, 771-784.

Cavallini E, Matus JT, Finezzo L, Zenoni S, Loyola R, Guzzo F, Schlechter R, Ageorges A, Arce-Johnson P, Tornielli GB. 2015. The Phenylpropanoid Pathway Is Controlled at Different Branches by a Set of R2R3-MYB C2 Repressors in Grapevine. *Plant Physiology* **167**, 1448-1470.

Cedroni ML, Cronn RC, Adams KL, Wilkins TA, Wendel JF. 2003. Evolution and expression of MYB genes in diploid and polyploid cotton. *Plant Molecular Biology* **51**, 313-325.

Colquhoun TA, Kim JY, Wedde AE, Levin LA, Schmitt KC, Schuurink RC, Clark DG. 2011. PhMYB4 fine-tunes the floral volatile signature of Petunia x hybrida through PhC4H. *Journal of Experimental Botany* **62**, 1133-1143.

Dubos C, Le Gourrierec J, Baudry A, Huep G, Lanet E, Debeaujon I, Routaboul JM, Alboresi A, Weisshaar B, Lepiniec L. 2008. MYBL2 is a new regulator of flavonoid biosynthesis in Arabidopsis thaliana. *Plant Journal* **55**, 940-953.

Fornale S, Lopez E, Salazar-Henao JE, Fernandez-Nohales P, Rigau J, Caparros-Ruiz D. 2014. AtMYB7, a new player in the regulation of UV-sunscreens in Arabidopsis thaliana. *Plant and Cell Physiology* **55**, 507-516.

Fornale S, Shi X, Chai C, Encina A, Irar S, Capellades M, Fuguet E, Torres JL, Rovira P, Puigdomenech P, Rigau J, Grotewold E, Gray J, Caparros-Ruiz D. 2010. ZmMYB31 directly represses maize lignin genes and redirects the phenylpropanoid metabolic flux. *Plant Journal* **64**, 633-644.

Gao JJ, Shen XF, Zhang Z, Peng RH, Xiong AS, Xu J, Zhu B, Zheng JL, Yao QH. 2011. The myb transcription factor MdMYB6 suppresses anthocyanin biosynthesis in transgenic Arabidopsis. *Plant Cell Tissue and Organ Culture* **106**, 235-242.

Huang YF, Vialet S, Guiraud JL, Torregrosa L, Bertrand Y, Cheynier V, This P, Terrier N. 2014. A negative MYB regulator of proanthocyanidin accumulation, identified through expression quantitative locus mapping in the grape berry. *New Phytologist* **201**, 795-809.

Jackson D, Culianez-Macia F, Prescott AG, Roberts K, Martin C. 1991. Expression patterns of myb genes from Antirrhinum flowers. *Plant Cell* **3**, 115-125.

Jun JH, Liu CG, Xiao XR, Dixon RA. 2015. The Transcriptional Repressor MYB2 Regulates Both Spatial and Temporal Patterns of Proanthocyandin and Anthocyanin Pigmentation in Medicago truncatula. *Plant Cell* **27**, 2860-2879.

Kiferle, C., Fantini, E., Bassolino, L., Povero, G., Spelt, C., Buti, S., Giuliano, G., Quattrocchio, F., Koes, R., Perata, P., and Gonzali, S. 2015, Tomato R2R3-MYB Proteins SlANT1 and SlAN2: Same Protein Activity, Different Roles. PLoS ONE **10**, e0136365.

Kirik V, Simon M, Huelskamp M, Schiefelbein J. 2004a. The ENHANCER OF TRY AND CPC1 gene acts redundantly with TRIPTYCHON and CAPRICE in trichome and root hair cell patterning in Arabidopsis. *Developmental Biology* **268**, 506-513.

Kirik V, Simon M, Wester K, Schiefelbein J, Hulskamp M. 2004b. ENHANCER of TRY and CPC 2 (ETC2) reveals redundancy in the region-specific control of trichome development of Arabidopsis. *Plant Molecular Biology* **55**, 389-398.

Kranz HD, Denekamp M, Greco R, Jin H, Leyva A, Meissner RC, Petroni K, Urzainqui A, Bevan M, Martin C, Smeekens S, Tonelli C, Paz-Ares J, Weisshaar B. 1998. Towards functional characterisation of the members of the R2R3-MYB gene family from Arabidopsis thaliana. *Plant Journal* **16**, 263-276.

Kroon A. 2004. Transcription Regulation of the Anthocyanin Pathway in Petunia hybrida. *PhD dissertation (Amsterdam: Vrije Universiteit).*

Larkin MA, Blackshields G, Brown NP, Chenna R, McGettigan PA, McWilliam H, Valentin F, Wallace IM, Wilm A, Lopez R, Thompson JD, Gibson TJ, Higgins DG. 2007, Clustal W and Clustal X version 2.0. Bioinformatics **23**, 2947-2948

Legay S, Sivadon P, Blervacq AS, Pavy N, Baghdady A, Tremblay L, Levasseur C, Ladouce N, Lapierre C, Seguin A, Hawkins S, Mackay J, Grima-Pettenati J. 2010. EgMYB1, an R2R3 MYB transcription factor from eucalyptus negatively regulates secondary cell wall formation in Arabidopsis and poplar. *New Phytologist* **188**, 774-786.

Lin-Wang K, Micheletti D, Palmer J, Volz R, Lozano L, Espley R, Hellens RP, Chagne D, Rowan DD, Troggio M, Iglesias I, Allan AC. 2011. High temperature reduces apple fruit colour via modulation of the anthocyanin regulatory complex. *Plant Cell and Environment* **34**, 1176-1190.

Liu YF, Li QT, Lu X, Song QX, Lam SM, Zhang WK, Ma B, Lin Q, Man WQ, Du WG, Shui GH, Chen SY, Zhang JS. 2014. Soybean GmMYB73 promotes lipid accumulation in transgenic plants. *BMC Plant Biology* **14**, 73.

Matsui K, Umemura Y, Ohme-Takagi M. 2008. AtMYBL2, a protein with a single MYB domain, acts as a negative regulator of anthocyanin biosynthesis in Arabidopsis. *Plant Journal* **55**, 954-967.

Nicholas KB, Nicholas HBJ, Deerfield DWI.1997. GeneDoc: analysis and visualization of genetic variation. EMBNEWNEWS **4**, 14.

Omer S, Kumar S, Khan BM. 2013. Over-expression of a subgroup 4 R2R3 type MYB transcription factor gene from Leucaena leucocephala reduces lignin content in transgenic tobacco. *Plant Cell Rep* **32**, 161-171.

Perez-Diaz JR, Perez-Diaz J, Madrid-Espinoza J, Gonzalez-Villanueva E, Moreno Y, Ruiz-Lara S. 2016. New member of the R2R3-MYB transcription factors family in grapevine suppresses the anthocyanin accumulation in the flowers of transgenic tobacco. *Plant Molecular Biology* **90**, 63-76.

Povero, G., Gonzali, S., Bassolino, L., Mazzucato, A., & Perata, P. 2011. Transcriptional analysis in high-anthocyanin tomatoes reveals synergistic effect of Aft and atv genes. Journal of plant physiology, **168(3)**, 270-279.

Preston J, Wheeler J, Heazlewood J, Li SF, Parish RW. 2004. AtMYB32 is required for normal pollen development in Arabidopsis thaliana. *Plant Journal* **40**, 979-995.

Qiu Z, Wang X, Gao J, Guo Y, Huang Z, Du Y. 2016. The Tomato *Hoffman's Anthocyaninless* Gene Encodes a bHLH Transcription Factor Involved in Anthocyanin Biosynthesis That Is Developmentally Regulated and Induced by Low Temperatures. PloS ONE **11**, e0151067.

Schnittger A, Folkers U, Schwab B, Jurgens G, Hulskamp M. 1999. Generation of a spacing pattern: the role of triptychon in trichome patterning in Arabidopsis. *Plant Cell* **11**, 1105-1116.

Simon M, Lee MM, Lin Y, Gish L, Schiefelbein J. 2007. Distinct and overlapping roles of single-repeat MYB genes in root epidermal patterning. *Developmental Biology* **311**, 566-578.

Sonbol FM, Fornale S, Capellades M, Encina A, Tourino S, Torres JL, Rovira P, Ruel K, Puigdomenech P, Rigau J, Caparros-Ruiz D. 2009. The maize ZmMYB42 represses the phenylpropanoid pathway and affects the cell wall structure, composition and degradability in Arabidopsis thaliana. *Plant Molecular Biology* **70**, 283-296.

Tang XF, Zhuang YM, Qi G, Wang D, Liu HH, Wang KR, Chai GH, Zhou GK. 2015. Poplar PdMYB221 is involved in the direct and indirect regulation of secondary wall biosynthesis during wood formation. *Scientific Reports* **5**, 12240.

Wada T, Tachibana T, Shimura Y, Okada K. 1997. Epidermal cell differentiation in Arabidopsis determined by a Myb homolog, CPC. *Science* **277**, 1113-1116.

Wan S, Li C, Ma X, Luo K. 2017. PtrMYB57 contributes to the negative regulation of anthocyanin and proanthocyanidin biosynthesis in poplar. *Plant Cell Rep*.**36**, 1263-1276.

Xu F, Ning YJ, Zhang WW, Liao YL, Li LL, Cheng H, Cheng SY. 2014. An R2R3-MYB transcription factor as a negative regulator of the flavonoid biosynthesis pathway in Ginkgo biloba. *Functional & Integrative Genomics* **14**, 177-189.

Yan JH, Wang B, Zhong YP, Yao LM, Cheng LJ, Wu TL. 2015. The soybean R2R3 MYB transcription factor GmMYB100 negatively regulates plant flavonoid biosynthesis. *Plant Molecular Biology* **89**, 35-48.

Yoshida K, Ma DW, Constabel CP. 2015. The MYB182 Protein Down-Regulates Proanthocyanidin and Anthocyanin Biosynthesis in Poplar by Repressing Both Structural and Regulatory Flavonoid Genes. *Plant Physiology* **167**, 693-710.

Yuan YW, Sagawa JM, Young RC, Christensen BJ, Bradshaw HD. 2013. Genetic Dissection of a Major Anthocyanin QTL Contributing to Pollinator-Mediated Reproductive Isolation Between Sister Species of Mimulus. *Genetics* **194**, 255-263.

Zhou H, Peng Q, Zhao JB, Owiti A, Ren F, Liao L, Wang L, Deng XB, Jiang Q, Han YP. 2016. Multiple R2R3-MYB Transcription Factors Involved in the Regulation of Anthocyanin Accumulation in Peach Flower. *Front Plant Sci* **7,** 1557.

Zhu L, Shan H, Chen S, Jiang J, Gu C, Zhou G, Chen Y, Song A, Chen F. 2013. The Heterologous Expression of the Chrysanthemum R2R3-MYB Transcription Factor CmMYB1 Alters Lignin Composition and Represses Flavonoid Synthesis in Arabidopsis thaliana. *PLoS One* **8**, e65680.
